# Vaccination with *Plasmodium vivax* Duffy-binding protein inhibits parasite growth during controlled human malaria infection

**DOI:** 10.1126/scitranslmed.adf1782

**Published:** 2023-07-12

**Authors:** Mimi M. Hou, Jordan R. Barrett, Yrene Themistocleous, Thomas A. Rawlinson, Ababacar Diouf, Francisco J. Martinez, Carolyn M. Nielsen, Amelia M. Lias, Lloyd D. W. King, Nick J. Edwards, Nicola M. Greenwood, Lucy Kingham, Ian D. Poulton, Baktash Khozoee, Cyndi Goh, Susanne H. Hodgson, Dylan J. Mac Lochlainn, Jo Salkeld, Micheline Guillotte-Blisnick, Christèle Huon, Franziska Mohring, Jenny M. Reimer, Virander S. Chauhan, Paushali Mukherjee, Sumi Biswas, Iona J. Taylor, Alison M. Lawrie, Jee-Sun Cho, Fay L. Nugent, Carole A. Long, Robert W. Moon, Kazutoyo Miura, Sarah E. Silk, Chetan E. Chitnis, Angela M. Minassian, Simon J. Draper

**Affiliations:** 1 Department of Biochemistry, University of Oxford; Oxford, OX1 3QU, UK; 2 The Jenner Institute, University of Oxford; Oxford, OX3 7DQ, UK; 3 Kavli Institute for Nanoscience Discovery, University of Oxford, Oxford, OX1 3QU, UK; 4 Laboratory of Malaria and Vector Research, NIAID/NIH; Rockville, MD 20852, USA; 5 Unité de Biologie de Plasmodium et Vaccins, Institut Pasteur, Université Paris Cité; 25-28 Rue du Dr Roux, 75015 Paris, France; 6 Department of Infection Biology, London School of Hygiene and Tropical Medicine; London, WC1E 7HT, UK; 7 Novavax AB; Kungsgatan 109, SE-753 18, Uppsala, Sweden; 8 International Centre for Genetic Engineering and Biotechnology (ICGEB); New Delhi, India; 9 Multi-Vaccines Development Program (MVDP); New Delhi, India; 10 NIHR Oxford Biomedical Research Centre, Oxford, UK

## Abstract

There are no licensed vaccines against *Plasmodium vivax*. We conducted two Phase I/IIa clinical trials to assess two vaccines targeting *P. vivax* Duffy-binding protein region II (PvDBPII). Recombinant viral vaccines using chimpanzee adenovirus 63 (ChAd63) and modified vaccinia virus Ankara (MVA) vectors, as well as a protein and adjuvant formulation (PvDBPII/Matrix-M) were tested in both a standard and delayed dosing regimen. Volunteers underwent controlled human malaria infection (CHMI) following their last vaccination, alongside unvaccinated controls. Efficacy was assessed by comparison of parasite multiplication rate in blood. PvDBPII/Matrix-M, given in a delayed dosing regimen, elicited the highest antibody responses and reduced the mean parasite multiplication rate following CHMI by 51% (n=6) compared to unvaccinated controls (n=13), whereas no other vaccine or regimen impacted parasite growth. Both viral-vectored and protein vaccines were well tolerated and elicited expected, short lived adverse events. Together, these results support further clinical evaluation of the PvDBPII/Matrix-M *P. vivax* vaccine.

## Introduction

*Plasmodium vivax* is the second most common cause of malaria and most geographically widespread, causing an estimated 4.5 million cases in 2020 (1). Control of *P. vivax* is more challenging than *P. falciparum* due to several factors. These include the ability of *P. vivax* to form dormant liver-stage hypnozoites that can reactivate and lead to relapsing blood-stage parasitemia, and earlier production of gametocytes in the blood-stage resulting in more rapid transmission (2). An effective vaccine would greatly aid elimination efforts worldwide but few *P. vivax* vaccines have reached clinical development.

Candidate vaccines against *P. vivax* have been developed that target different stages of the parasite’s lifecycle (3). These include blood-stage vaccines that aim to inhibit the invasion of reticulocytes by merozoites, the stage of infection causing clinical disease. The leading blood-stage vaccine target is *P. vivax* Duffy-binding protein (PvDBP), which binds to the Duffy antigen receptor for chemokines (DARC/Fy) on reticulocytes to mediate invasion of the parasite (4). This interaction is critical as evidenced by the natural resistance of Duffy antigen negative individuals to *P. vivax* malaria (5). However, the efficacy of blocking this molecular interaction with vaccine-induced antibodies has not been tested previously in clinical trials.

Two vaccines targeting region II of PvDBP (PvDBPII), a 327-amino acid domain that binds to DARC, have previously progressed to Phase I clinical trials. These vaccines comprise a recombinant viral-vectored chimpanzee adenovirus 63 (ChAd63)-modified vaccinia virus Ankara (MVA) platform (6) and a protein/adjuvant formulation (PvDBPII/GLA-SE) (7). Both vaccines encode the Salvador I (SalI) allele of PvDBPII and were shown to induce binding-inhibitory antibodies (BIA) that block the interaction of recombinant PvDBPII to the DARC receptor in vitro (6, 7).

Here we report results from two Phase I/IIa clinical trials in healthy malaria-naïve adults using either the same viral-vectored vaccine or the PvDBPII protein vaccine reformulated in Matrix-M adjuvant. Both vaccines were tested for efficacy for the first time by blood-stage controlled human malaria infection (CHMI) using the heterologous PvW1 clone of *P. vivax* (8).

## Results

### Participants and trial design

**Figure 1:**
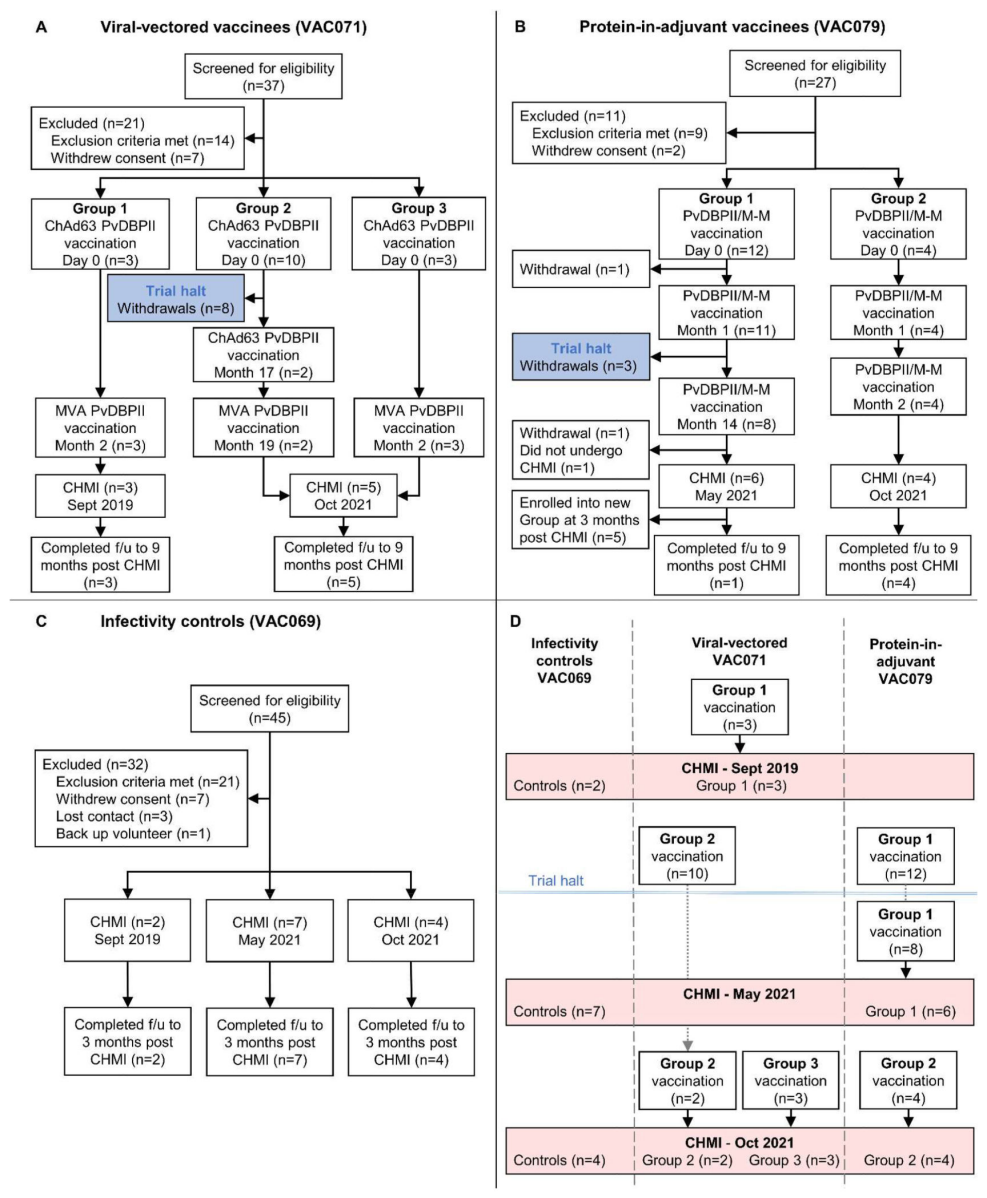
Flow charts of study design and participant recruitment. (**A**) VAC071 Group 1 participants received the viral-vectored vaccines ChAd63 PvDBPII and MVA PvDBPII 8 weeks apart, followed by CHMI 2 to 4 weeks later. Group 2 received ChAd63 PvDBPII before the trial was temporarily halted. On restart of the trial, returning participants in Group 2 received a second dose of ChAd63 PvDBPII at 17 months, followed by MVA PvDBPII 8 weeks later. Group 3 participants received the 8-week viral-vectored vaccine regimen and underwent CHMI along with Group 2 volunteers at 2 to 4 weeks after the final vaccination. (**B**) VAC079 participants received protein PvDBPII vaccine in Matrix-M adjuvant (PvDBPII/M-M). Group 1 volunteers received three doses at 0-1-14 months (delayed third dose due to trial halt). Group 2 volunteers received three doses at 0-1-2 months, with CHMI at 2 to 4 weeks after the final vaccination. (**C**) VAC069 participants underwent blood-stage CHMI in three separate stages and acted as infectivity controls for vaccinees undergoing CHMI in parallel. (**D**) Shown is a summary of the three CHMIs. VAC071 Group 1 vaccinees underwent CHMI in parallel with control participants in September 2019. In January 2020 vaccinations commenced in VAC071 and VAC079, before the trials were halted in March 2020. After restart of the VAC079 trial in 2021, Group 1 participants underwent CHMI in parallel with control participants in May 2021. In October 2021, control participants underwent CHMI in parallel with vaccinees from VAC071 Groups 2 and 3 and VAC079 Group 2. f/u, follow-up.

Sixteen volunteers were enrolled into the VAC071 trial testing the viral-vectored vaccines (VV-PvDBPII) between July 2019 and July 2021 (Fig. 1A). Three volunteers in Group 1 received ChAd63 followed by MVA PvDBPII at 0 and 2 months. Ten volunteers in Group 2 received ChAd63 PvDBPII in February 2020, prior to the trial being halted due to the coronavirus disease 2019 (COVID-19) pandemic. After restart of the trial, two of the ten volunteers were re-enrolled and received a second dose of ChAd63 PvDBPII at 17 months, followed by MVA PvDBPII at 19 months. Three volunteers enrolled into Group 3 received one dose of ChAd63 followed by MVA PvDBPII at 0 and 2 months. Vaccinees underwent CHMI 2 to 4 weeks after their final vaccination.

Sixteen volunteers were enrolled into the VAC079 trial testing the protein vaccine PvDBPII in Matrix-M adjuvant (PvDBPII/M-M) between January 2020 and July 2021 (Fig. 1B). Twelve volunteers enrolled into Group 1 in 2020 received two doses of PvDBPII/M-M at 0 and 1 months before the trial was halted due to the COVID-19 pandemic. After restart of the trial in 2021, eight of the twelve volunteers were re-enrolled and received a third vaccination at 14 months and six of these volunteers underwent CHMI 2 to 4 weeks later. Four volunteers enrolled into Group 2 in July 2021 received three doses of PvDBPII/M-M at 0, 1 and 2 months, followed by CHMI 2 to 4 weeks later.

Thirteen infectivity control volunteers underwent CHMI in parallel with vaccinees over three phases of the VAC069 study (Fig. 1C and D). Demographics of volunteers in each trial are provided in table S1. Control volunteers were followed-up to 3 months post-CHMI. Vaccinees were followed-up to 9 months post-CHMI, apart from i) the final study visits for Group 1 volunteers in VAC071 fell during the trial halt and were conducted remotely by telephone without phlebotomy; and ii) five out of six Group 1 volunteers from the VAC079 trial who completed CHMI were enrolled into a new study group at 3 months post-CHMI and their data after this timepoint are not reported here.

### Vaccine safety

**Figure 2:**
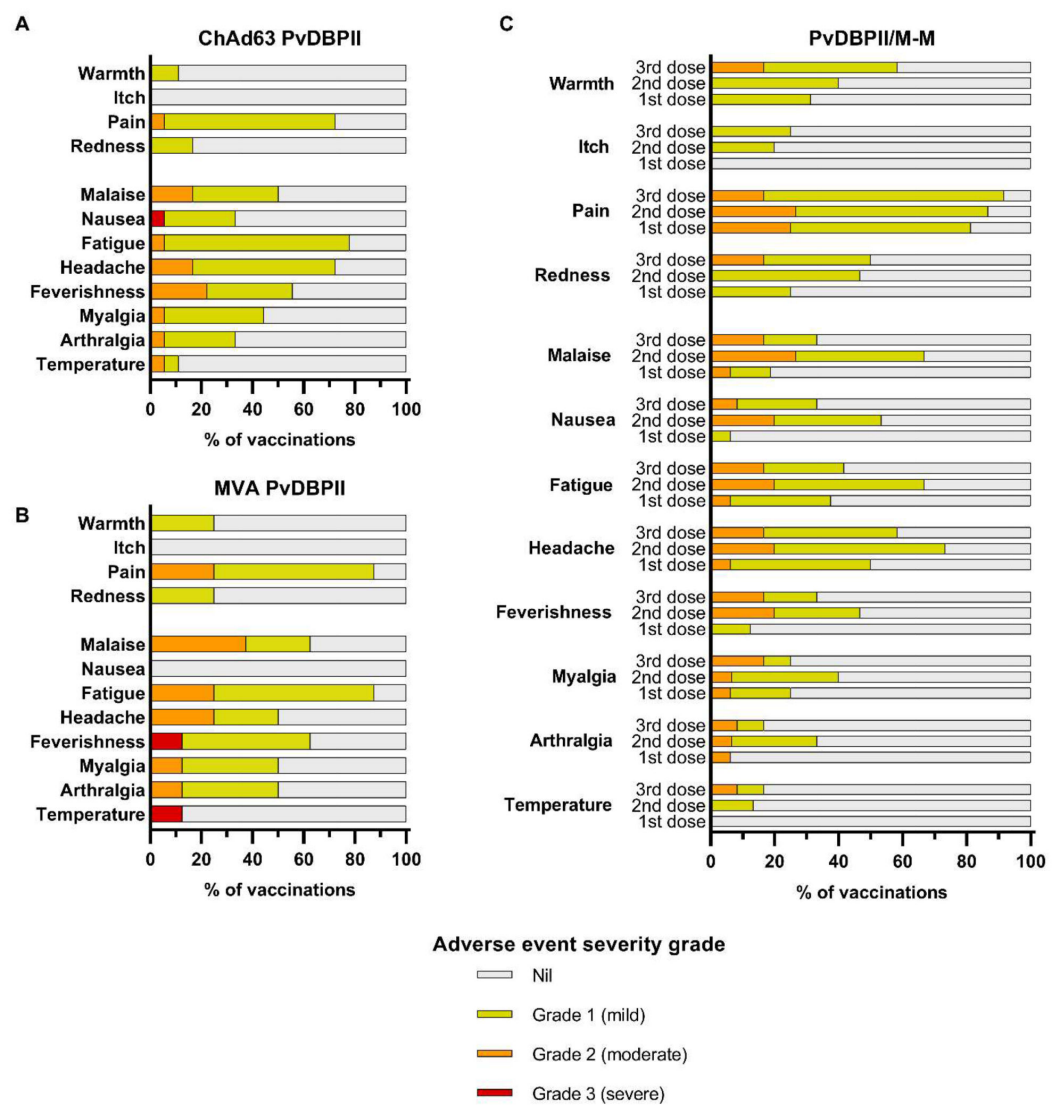
Local and systemic solicited adverse events. Solicited AEs were recorded by volunteers within 7 days following each vaccination in participant symptom electronic diaries. The maximal severity reported for each AE is shown as a percentage of the number of vaccinations administered. (**A**) ChAd63 PvDBPII AEs are shown; n=18 vaccinations (16 volunteers received one dose, 2 volunteers received a second dose). (**B**) MVA PvDBPII AEs are shown; n=8 vaccinations (8 volunteers received one dose). (**C**) PvDBPII/M-M AEs are shown. AEs reported after first (n=16), second (n=15), and third doses (n=12) are shown.

No safety concerns were identified with the viral-vectored or protein-in-adjuvant vaccines and no serious adverse events (AE) occurred in the VAC071 and VAC079 trials. The viral-vectored vaccines showed similar reactogenicity to that previously reported (6). Solicited AEs were predominantly mild to moderate in severity, with pain at the injection site and fatigue being most common (Fig. 2A and B). Three severe solicited AEs occurred post-vaccination, all of which resolved within 48 hours: nausea in one individual following ChAd63 PvDBPII vaccination, and feverishness and pyrexia in another individual following MVA PvDBPII vaccination. Solicited AEs following vaccinations with PvDBPII/M-M were all mild to moderate in severity and no severe adverse events occurred (Fig. 2C). Injection site pain and headache were the most common solicited AEs. Transient lymphopenia, with maximal severity of grade 2, occurred commonly following vaccinations with both the viral-vectored and protein-in-adjuvant vaccines (table S2). Unsolicited AEs deemed at least possibly related to either viral-vectored or protein-in-adjuvant vaccinations were of mild to moderate severity and self-limited (tables S3 and S4).

### Viral-vectored and protein PvDBPII vaccines elicited antibody responses

**Figure 3:**
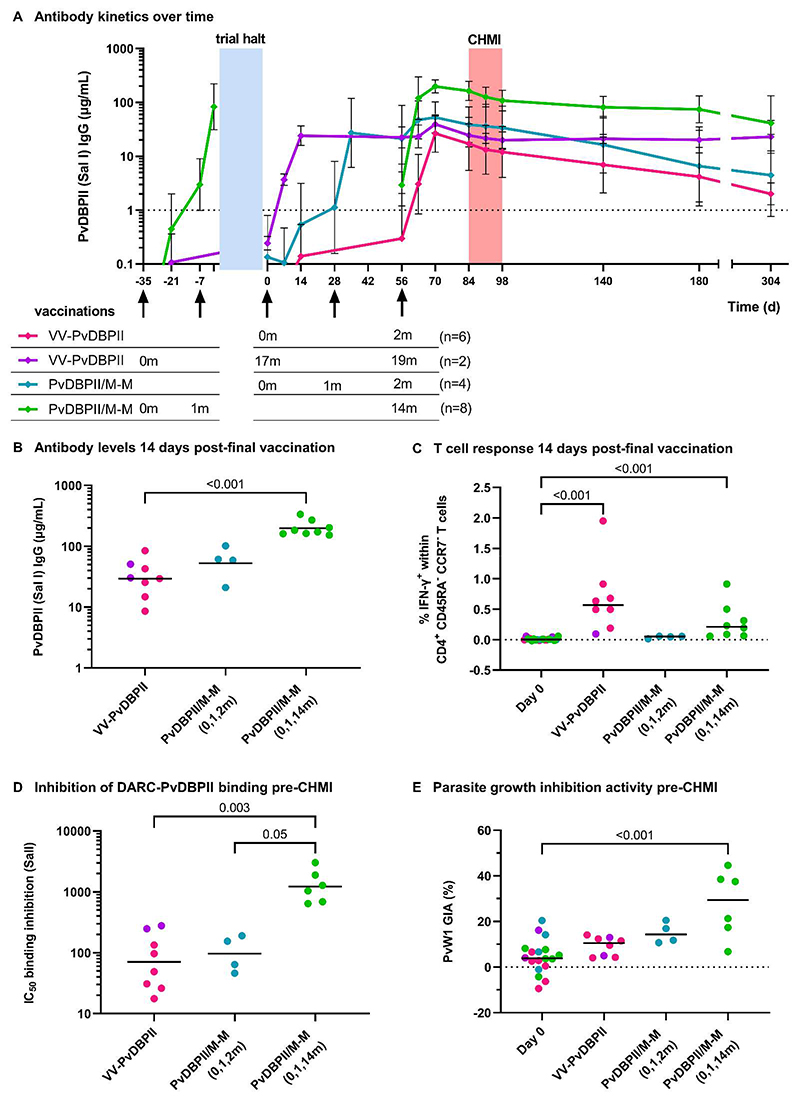
Immunological responses were elicited by PvDBPII vaccinations. (**A**) Anti-PvDBPII Salvador I (SalI) strain total IgG serum concentrations are shown over time for each vaccination regimen showing geometric mean with standard deviation. Groups are aligned at the time of final vaccination (day 56). Arrows indicate vaccinations with timing of doses in each regimen indicated below in months. VV-PvDBPII indicates viral-vectored vaccines. Blue shading indicates trial halt of about 1 year, vaccinations occurring prior to the trial halt are shown to the left. Red shading indicates period of controlled human malaria infection (CHMI). IgG concentrations below 1 μg/mL, indicated by dotted line, are classified as negative responses but shown for clarity. (**B**) Shown are individual anti-PvDBPII (SalI) total IgG serum concentrations 14 days post-final vaccination with geometric means for each regimen. (**C**) Shown are the percentages of IFN-γ^+^ cells within CD4^+^ CD45RA^-^ CCR7^-^ effector memory T cells collected 14 days post-final vaccination following stimulation of peripheral blood mononuclear cells with a pool of PvDBPII (SalI) peptides, with group medians. The frequency of IFN-γ^+^ cells in sample-matched unstimulated wells was subtracted to control for non-specific activation. Baseline responses (Day 0) are shown for all volunteers. (**D**) Shown are the dilution factors of individual serum, taken pre-CHMI, required to inhibit DARC-PvDBPII (SalI) binding by 50% (IC_50_) with geometric means. Baseline responses (Day 0) are shown for all volunteers. (**E**) Shown is the percentage of in vitro growth inhibition activity (GIA) of 10 mg/mL purified total IgG, taken pre-CHMI, against *P. knowlesi* parasites expressing PvDBP PvW1 allele, with medians. Baseline responses (Day 0) are shown for all volunteers. *p* values were calculated by Kruskal-Wallis test with Dunn’s multiple comparison post-test.

Anti-PvDBPII (SalI) total IgG serum antibody responses peaked around 2 weeks following the final vaccination in all regimens (Fig. 3A). PvDBPII/M-M given at 0, 1, and 14 months induced the highest antibody response at this timepoint (geometric mean 198 μg/mL [range 153 to 335]), which was significantly higher than the viral-vectored vaccines (29 μg/mL [range 9 to 85]; *p*<0.001) (Fig. 3B). Anti-PvDBPII antibody responses were negative (less than 1 μg/mL) in all vaccinees prior to their first vaccination, and in controls remained below 1 μg/mL throughout. Antibody responses waned relatively quickly from their peak during the first month, with no boosting observed during CHMI, followed by a slower rate of decline which plateaued in some volunteers by 10 months post-final vaccination. Antibody longevity for each individual was estimated by calculating the area under the curve (AUC) from the time of the peak antibody concentration to the final timepoint available, divided by the peak antibody concentration and duration over which the AUC was calculated. Antibody longevity did not differ between different dosing regimens (fig. S1).

PvDBPII-specific CD4^+^ CD45RA^-^ CCR7^-^ effector memory T cells producing interferon (IFN)-γ were detectable following final vaccinations with VV-PvDBPII and PvDBPII/M-M administered in a delayed dosing regimen (Fig. 3C). IFN-γ producing CD8^+^ effector memory T cells were observed at low frequencies in the VV-PvDBPII vaccinees and were not detectable in the protein vaccine groups (fig. S2 and S3, table S5).

Serum taken pre-CHMI from vaccinees administered PvDBPII/M-M in the delayed dosing regimen showed roughly 10-fold higher BIA values (geometric mean of dilution factor to achieve 50% binding inhibition (IC_50_) = 1224 [range 643 to 3026]) as compared to the monthly dosing regimen and VV-PvDBPII (Fig. 3D). Using the dilution factor and total IgG concentration in the serum, the concentration of anti-PvDBPII total IgG that is required to achieve 50% binding inhibition was calculated for each individual. This was lower in the PvDBPII/M-M delayed dosing regimen group (median 123 ng/mL [range 78 to 250]) as compared to the monthly regimen of PvDBPII/M-M (447 ng/mL [196-715]; *p*=0.05) (fig. S4). The binding inhibition IC_50_ in the VV-PvDBPII group did not differ from the other groups when all 8 vaccinees’ data were combined but the two vaccinees who received a second dose of ChAd63 PvDBPII after the trial halt had a low binding inhibition IC_50_ of 62 and 140 ng/mL.

Functional anti-parasitic in vitro growth inhibition activity (GIA) was generally low pre-CHMI. The highest activity was observed in the PvDBPII/M-M delayed dosing regimen with median GIA of 29% (range 7 to 45%) against transgenic *P. knowlesi* expressing the PvDBP PvW1 allele (challenge sequence) (Fig. 3E). GIA against *P. knowlesi* expressing the PvDBP SalI allele (vaccine sequence) were similar and correlated well with GIA against the PvW1 allele (fig. S5A and B). Serum IgG responses and BIA assayed using the PvW1 sequence of PvDBPII were also well correlated and in concordance with responses to the SalI PvDBPII sequence (Fig. S5C to E). BIA correlated strongly with anti-PvDBPII total IgG serum antibody responses measured by ELISA, whilst GIA versus ELISA indicated the start of a sigmoidal relationship, as previously seen with *P. falciparum* blood-stage vaccines (9) (fig. S6).

### Delayed PvDBPII/M-M vaccination slowed parasite multiplication rate after CHMI

**Figure 4:**
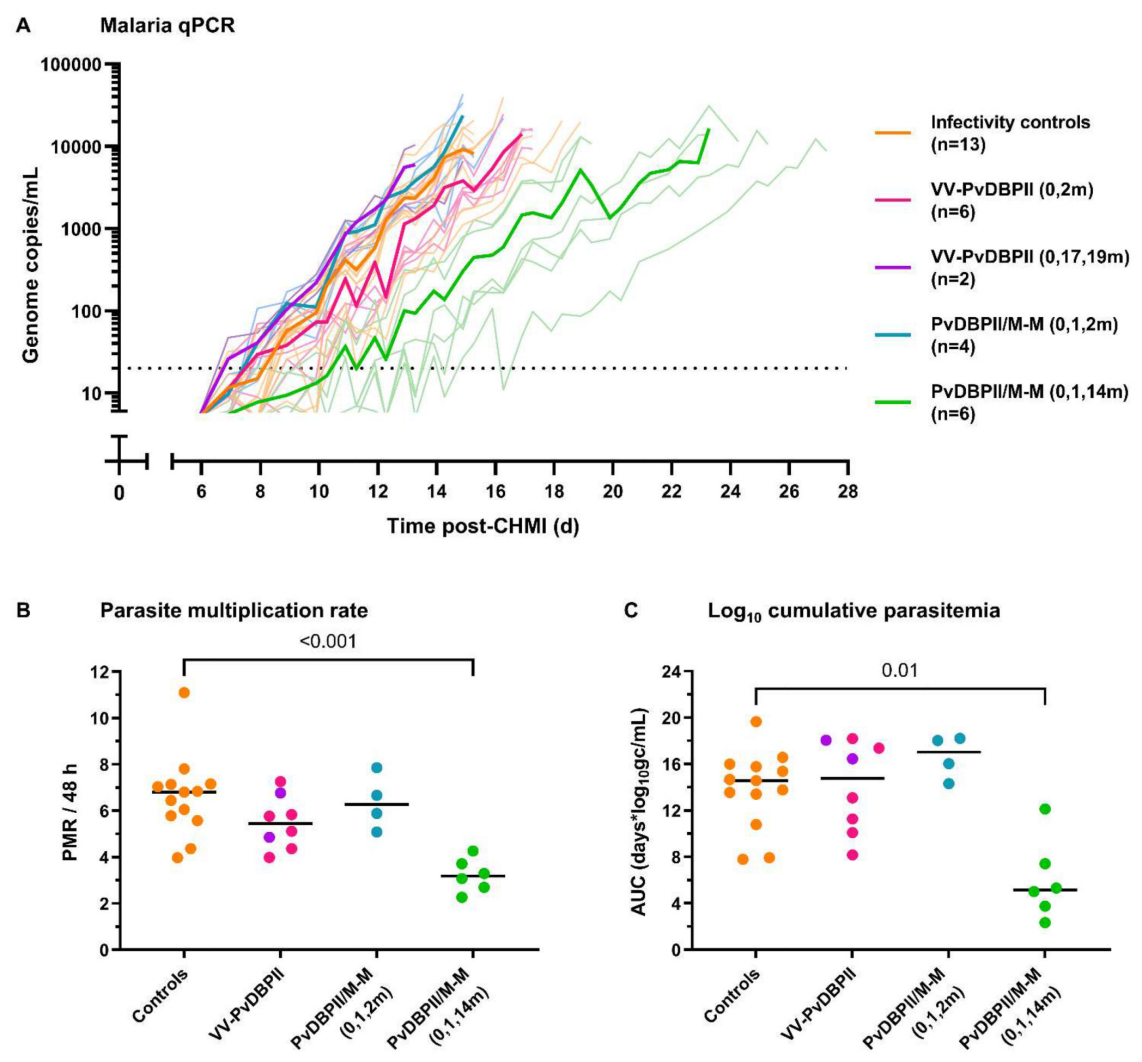
PvDBPII/M-M inhibits growth of *P. vivax* after CHMI. (**A**) Individual parasitemia over time was measured by qPCR, with group means in bold lines. Timings of vaccinations are shown in brackets in months. On the day of CHMI, volunteers were administered an intravenous injection of *P. vivax* (PvW1 clone) blood-stage parasites. The dotted line indicates the minimum concentration of parasitemia to meet positive reporting criteria (20 genome copies [gc]/mL). (**B**) Shown is a comparison of parasite multiplication rate (PMR) per 48 hours between vaccinees and controls. Individual PMRs are modelled from the qPCR data over time and are shown with group median. (**C**) Shown is a comparison of log_10_ cumulative parasitemia (LCP) during CHMI between vaccinees and controls with group median. LCP calculated from area under the curve (AUC) of log_10_-transformed qPCR over time for each individual, up until day 14 after challenge when the first volunteer reached malaria diagnostic criteria across all CHMIs. *p* values were calculated by Kruskal-Wallis test with Dunn’s multiple comparison post-test.

Following blood-stage CHMI with the heterologous PvW1 clone of *P. vivax*, all volunteers developed parasitemia and received antimalarial treatment after reaching protocol specified malaria diagnostic criteria (Fig. 4A, tables S6 to S8). Volunteers administered the PvDBPII/M-M vaccine, but not VV-PvDBPII, had significantly lower parasite multiplication rate (PMR) as compared to controls (table S9, *p*=0.01). Post-hoc analysis showed that this was due to the delayed dosing regimen group of PvDBPII/M-M, who had a significantly lower median PMR of 3.2-fold growth per 48 hours (range 2.3 to 4.3) compared to the unvaccinated controls (median PMR of 6.8-fold growth per 48 hours [range 4.0 to 11.1], *p*<0.001) (Fig. 4B). This equated to a 53% reduction in median PMR and was reflected in a 7-day delay in median time to reach malaria diagnosis, from 15.5 days in controls to 22.5 days in vaccinees (fig. S7). Exploratory analysis of log_10_ cumulative parasitemia (LCP) gave concordant results and showed significantly lower LCP in those administered PvDBPII/M-M in the delayed dosing regimen as compared to controls (Fig. 4C, *p*=0.01). PMR and LCP significantly correlated (fig. S8, *p*=0.01). The other vaccination regimens showed no impact on any outcome measure. PMR did not differ by Duffy blood group serophenotype, after adjusting for vaccination group (table S10). Parasitemia at the time of malaria diagnosis was consistent across all groups (fig. S9). The frequency and severity of solicited malaria symptoms, pyrexia and hematological and biochemical laboratory abnormalities occurring during CHMI were similar between vaccinees and the control volunteers (fig. S10).

### Antibody readouts after vaccination correlated with in vivo parasite growth inhibition

**Figure 5:**
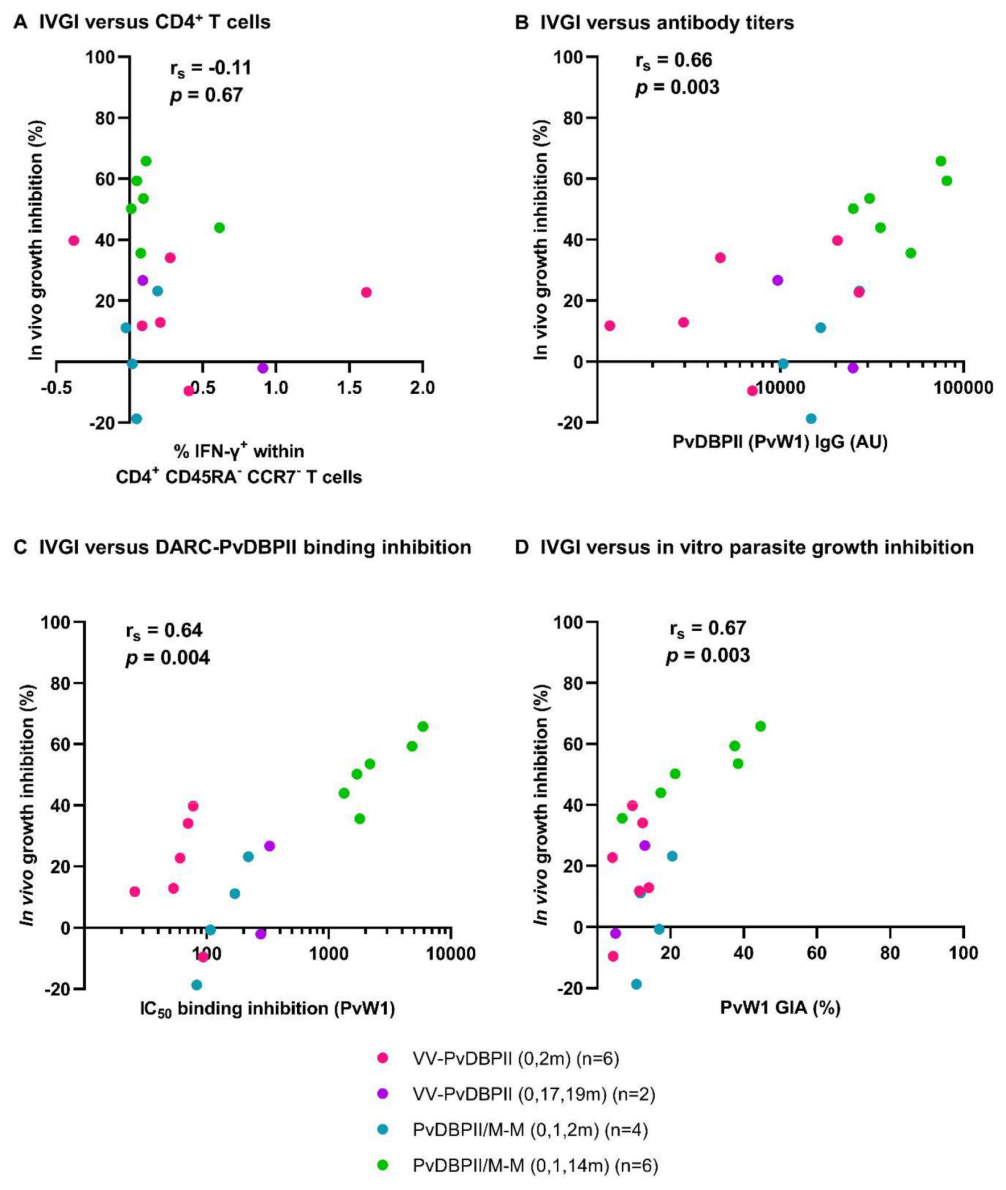
Antibody activity correlates with in vivo parasite growth inhibition. (**A to D**) The percent of in vivo parasite growth inhibition (IVGI), calculated as % reduction in PMR in vaccinees relative to the mean PMR in infectivity controls were correlated with pre-CHMI measurements of percentage of IFN-γ^+^ cells within CD4^+^ CD45RA^-^ CCR7^-^ effector memory T cells (**A**); anti-PvDBPII (PvW1) total IgG serum titers in arbitrary units (AU) (**B**); dilution factor of individual serum required to inhibit DARC-PvDBPII (PvW1) binding by 50% (IC_50_) (**C**); and (**D**) % in vitro GIA of 10 mg/mL purified total IgG against *P. knowlesi* parasites expressing the PvDBP PvW1 allele. Spearman’s rank correlation coefficients and *p* values are shown, n=18.

We assessed the relationship between measurements of vaccine immunogenicity pre-CHMI with in vivo growth inhibition (IVGI) observed during CHMI. IVGI was calculated for each vaccinated individual as the percentage reduction in PMR relative to the mean PMR in the unvaccinated controls. The mean IVGI in those administered PvDBPII/M-M in the delayed dosing regimen was 51% (range 36% to 66%). We found no association between IVGI and vaccine-induced CD4^+^ T cell IFN-γ responses (Fig. 5A). In contrast, correlations were observed between IVGI and all three antibody readouts: anti-PvDBPII (PvW1) total IgG serum antibody enzyme-linked immunosorbent assay (ELISA; Fig. 5B), BIA using PvW1 sequence PvDBPII protein (Fig. 5C), and in vitro GIA using purified IgG against *P. knowlesi* parasites expressing the PvDBP PvW1 allele (Fig. 5D).

## Discussion

The interaction between PvDBP and its host receptor DARC/Fy is critical for *P. vivax* invasion of reticulocytes, which explains the natural resistance of Duffy-negative individuals to *P. vivax* blood-stage infection (5). Structural studies have demonstrated that region II within PvDBP binds to DARC (10) and numerous immuno-epidemiological studies (11, 12) and preclinical vaccine models (13, 14) have supported the hypothesis that vaccine-induced anti-PvDBPII antibodies could inhibit blood-stage *P. vivax* parasite growth. Here we present the clinical vaccine trial results confirming this concept.

The Phase I/IIa trials reported here tested two different vaccine platforms to deliver the PvDBPII antigen. Results indicated no safety concerns and both vaccine formulations induced immune responses to PvDBPII. However, following CHMI, only the protein-in-adjuvant vaccine PvDBPII/M-M, given in a delayed 0-1-14 month dosing regimen, inhibited parasite growth. The average reduction of parasite growth by 51% confirms that vaccines targeting PvDBPII can induce anti-parasitic immunity. In comparison, the most advanced blood-stage *P. falciparum* vaccine, RH5.1/AS01_B_, achieved much higher in vitro GIA but reduced parasite growth in vivo by only around 20% following CHMI (9). The reduction in parasite growth in those who received PvDBPII/M-M in the delayed dosing regimen was reflected in a 7-day delay to reach malaria diagnosis and associated delay in development of malaria symptoms. However, there was no reduction in the severity of clinical malaria in these vaccinees compared to controls once they reached the protocol specified malaria diagnostic criteria.

Parasite growth rate during CHMI was calculated from the slope of a linear model fitted to log_10_ transformed quantitative polymerase chain reaction (qPCR) data, the method we have used for blood-stage CHMI studies to date (8, 9, 15, 16). PMR is based only on the rate of parasite growth after parasitemia is detectable by qPCR and does not differentiate between differences in time to reach detectable parasitemia. We therefore also calculated log_10_ cumulative parasitemia, a potentially more differentiating measure as it is affected by both the time to reach detectable parasitemia and the rate of parasite growth. In the future, more effective vaccines could control and clear parasitemia and result in non-linear parasite growth, in which case PMR may also not be the most appropriate metric to measure the degree of parasite growth. Of note the number of parasites administered during blood-stage CHMI are log_10_-fold smaller than the number of merozoites that are estimated to emerge from the liver during natural infection, therefore a longer time to patent parasitemia may be seen following CHMI as compared to natural infection. Nevertheless, the blood-stage CHMI model provides a robust means to detect relatively small reductions in PMR, which can be useful in identifying vaccine candidates with partial efficacy during early stages of clinical development (15).

A previous study has suggested that CD8^+^ T cells are able to kill *P. vivax* infected reticulocytes (17). In our vaccine trials, neither vaccine formulation induced a substantial antigen-specific IFN-γ^+^ CD8^+^T cell response. IFN-γ^+^ CD4^+^ T cell responses to vaccinations were observed, but there was no association between the magnitude of the response with parasite growth during CHMI. We also observed no effect of the Duffy blood group serophenotype on parasite multiplication rate, contrary to reports from field studies (18), although the number of volunteers in our studies was small. In contrast, our results indicate that the observed anti-parasitic immunity is antibody mediated, as evidenced by the association between in vivo parasite growth inhibition and three in vitro readouts of vaccine-induced antibodies: anti-PvDBPII-specific responses (measured by ELISA and functional BIA) and anti-parasitic GIA. These data provide important new benchmarks that link these assay readouts with in vivo outcome. The vaccine-induced in vitro GIA observed in these trials are modest, with median GIA of 29% with 10 mg/mL total IgG in the delayed dosing PvDBPII/M-M group. This is in contrast to much higher GIA achieved recently with the blood-stage P. falciparum vaccine RH5.1/AS01_B_, where median in vitro GIA of about 70% (range of about 60 to 90%) were observed in vaccinated healthy UK adults (9).

Our results also indicate that substantial gains in vaccine-induced antibodies can be achieved through modulation of delivery regimen. The delayed 0-1-14 month dosing regimen with PvDBPII/M-M showed improved immunogenicity, which translated into greater efficacy, as compared to the identical vaccine given in a 0-1-2 month regimen. The delayed dosing regimen in this study induced higher peak PvDBPII-specific antibody titers and more potent antibodies as measured by in vitro BIA and GIA, compared to monthly dosing of the PvDBPII/M-M vaccine. The greater binding inhibition may be due to improved antibody avidity, as has also been observed in the delayed dosing regimen of the *P. Falciparum* vaccine RH5.1/AS01_B_ (9). Further immunological analyses from that study suggested that the mechanism for the improvement was greater somatic hypermutation in B cell receptors in the delayed dosing group as compared to the monthly dosing group (19) and similar mechanisms could be acting in in the study reported here. Overall our data add to growing evidence that delayed dosing can improve vaccine-induced antibody responses, as has been seen with a variety of vaccine delivery technologies including those targeting *P. falciparum* and severe acute respiratory syndrome coronavirus 2 (SARS-CoV-2) (9, 20–22). However, the very long interval between vaccinations in the 0-1-14 month regimen used in this study would be difficult to implement in the field. The effectiveness of delayed dosing regimens with shorter intervals, such as 0-1-6 months, that could be more easily deployed should now be tested, and in vitro GIA could be used as a surrogate for in vivo outcome to systematically screen a variety of dosing regimens.

Apart from direct inhibition of ligand-receptor binding, vaccine-induced antibodies may mediate inhibition of parasites in vivo through Fc receptor-mediated functions, which are not measured by the standardized GIA assay. Further immunological analyses on larger cohorts to measure a variety of antibody functions could help determine which features in the delayed dosing regimen group are associated with in vivo growth inhibition. This includes measurements of antibody avidity and affinity, the quantity of major anti-PvDBPII specific antibody isotypes and subclasses and the capacity of antibodies to bind Fc receptors and activate different effector cells and complement. In a systems serology analysis of the *P. falciparum* RH5.1/AS01_B_ vaccine trial, the anti-RH5 IgA1 response was associated with challenge outcome (9). This suggests that other vaccine-induced immune mechanisms, apart from inhibition of erythrocyte invasion by IgG as measured by the GIA assay, may be acting to inhibit merozoites and could explain the only moderate correlation of GIA with in vivo parasite inhibition observed in the studies reported here.

A limitation of our trials is the small number of volunteers in each vaccination group due to withdrawals that occurred during the roughly 1 year trial halt secondary to the COVID-19 pandemic, which also necessitated changes to the vaccination regimens partway through the trials. After withdrawals of volunteers during the trial halt, the six volunteers who remained in the study and received the delayed dosing regimen of PvDBPII/M-M followed by CHMI were all female. This may be a confounding factor if females responded better than males to vaccination.

Another limitation is that our studies only used a single clone of *P. vivax* (PvW1) to assess vaccine efficacy. However, the PvW1 clone was recently isolated from a patient in Thailand and thus represents a currently circulating isolate (8). It also provided a heterologous challenge to the vaccine-induced responses raised against the SalI allele of PvDBPII. PvDBPII is highly polymorphic with distinct polymorphisms found in parasites from different geographical locations. The 10 polymorphisms in the PvDBP PvW1 allele as compared to the SalI allele are mostly non-conservative amino acid changes and are all found at positions at which polymorphisms occur at high frequency worldwide, including in the highly variant immunodominant ‘DEK’ epitope (23). In the studies reported here binding inhibition and GIA to the PvW1 allele of PvDBPII were well correlated with responses to the SalI allele. Along with the efficacy results, these data indicate that human immunization with PvDBPII can raise antibodies that recognize conserved epitopes within diverse PvDBPII variants. It will nonetheless be important for future studies to test the efficacy of PvDBPII-based vaccines against other heterologous *P. vivax* strains from different geographic locations, strains with PvDBP gene copy number variation (24), and parasites that infect Duffy-negative individuals (25).

Overall, this study represents an advance for the *P. vivax* blood-stage malaria vaccine field by confirming that vaccine-induced anti-PvDBPII immune responses can impact *P. vivax* growth in malaria-naïve individuals in vivo. Next steps will include CHMI or field efficacy trials of PvDBPII/M-M in malaria-endemic populations. Previous studies have shown that individuals in *P. vivax* endemic areas can acquire anti-PvDBPII antibody responses with increasing exposure, although only a minority of individuals develop high PvDBPII-DARC binding inhibitory antibody titers (11). Higher binding inhibition antibodies have been associated with delay in time to re-infection (11) and lower parasitemia during re-infection (12, 26). Vaccination with PvDBPII may enhance these pre-existing anti-malarial antibody responses in endemic populations or alternatively pre-existing anti-PvDBP antibodies may inhibit the response to vaccination.

In parallel, avenues to improve vaccine efficacy should be explored. Given that both of the PvDBPII vaccine candidates tested here were designed over 10 years ago, there is potential to rationally improve PvDBP vaccine immunogen design. Further studies to identify the epitopes or regions within this vaccine that elicit the most potent, strain-transcending antibodies will inform which responses need to be elicited in future vaccines (27). Approaches to focus the vaccine immune response include retaining only the most potent region of the vaccine immunogen or masking variant immunodominant epitopes that elicit only strain-specific responses (28). Use of newer and potentially more immunogenic vaccine platforms such as virus-like particles or mRNA may also improve vaccine efficacy. Looking beyond PvDBP, identifying new blood-stage antigen combinations that can elicit higher GIA (29, 30) and combining blood-stage vaccine with those targeting other lifecycle stages (31, 32) will likely be required to achieve high vaccine efficacy. Our data reported here provide the framework, with defined benchmark values of anti-PvDBPII antibodies and GIA versus IVGI, to guide rational design and delivery of next-generation blood-stage vaccines to protect against *P. vivax* malaria.

## Materials and Methods

### Study Design

Two Phase I/IIa vaccine efficacy trials (VAC071, VAC079) and a CHMI trial (VAC069) were conducted in parallel at a single site in the UK (Centre for Clinical Vaccinology and Tropical Medicine, University of Oxford). VAC071 was an open label trial to assess the ChAd63 and MVA viral-vectored vaccines encoding PvDBPII (VV-PvDBPII) (ClinicalTrials.gov number NCT04009096). VAC079 was also an open label trial and assessed the protein vaccine PvDBPII in Matrix-M adjuvant (PvDBPII/M-M) (ClinicalTrials.gov number NCT04201431). Unvaccinated infectivity controls were enrolled into the VAC069 trial (ClinicalTrials.gov number NCT03797989). Eligible volunteers were healthy, Duffy-positive, malaria-naïve adults, aged 18 to 45 years in the vaccine trials and 18 to 50 years in the VAC069 trial. Full volunteer inclusion and exclusion criteria are found in the supplementary methods. The original planned sample size was 15 volunteers for each vaccine trial. Vaccinations were interrupted in 2020 due to the COVID-19 pandemic. Following the trial halt of around 1 year and withdrawal of volunteers during this period, the trial was amended to allow completion of vaccinations of returning volunteers and to enroll new volunteers to undergo the original vaccination regimens. Vaccinees who completed their vaccinations regimens underwent CHMI at 2 to 4 weeks after their final vaccination, in parallel with infectivity controls from the VAC069 trial. Final sample sizes of volunteers undergoing CHMI were lower than planned due to withdrawals during the COVID-19 pandemic and difficulty with recruitment. The primary objective in both vaccine trials was to determine the efficacy of the vaccines by comparing the PMR during CHMI in vaccinees to the PMR in infectivity controls. Secondary objectives were to assess the safety and humoral and cellular immunogenicity of the vaccines and determine immunological readouts for association with a reduced parasite multiplication rate.

### Trial oversight

The trials were designed and conducted at the University of Oxford and received ethical approval from UK National Health Service Research Ethics Services. The VAC071 and VAC079 vaccine trials were approved by the UK Medicines and Healthcare products Regulatory Agency. All participants provided written informed consent and the trials were conducted according to the principles of the current revision of the Declaration of Helsinki 2008 and ICH guidelines for Good Clinical Practice.

### Vaccines

ChAd63 PvDBPII is a recombinant replication-defective chimpanzee adenovirus serotype 63 and MVA PvDBPII is a modified vaccinia virus Ankara vector, both encoding PvDBPII (SalI allele) (6). Recombinant PvDBPII protein (SalI allele) was produced in *Escherichia coli* to Good Manufacturing Practices at Syngene International (7). Matrix-M is a saponin-based adjuvant provided by Novavax AB which is licensed for use in their COVID-19 vaccine (Nuvaxovid). All vaccinations were administered intramuscularly into the deltoid muscle. ChAd63 PvDBPII was administered at a dose of 5x10^10^ viral particles (vp); MVA PvDBPII was administered at a dose of 2x10^8^ plaque forming units (pfu); and PvDBPII protein was administered at 50 μg, mixed with 50 μg Matrix-M. In the VAC071 trial, ChAd63 PvDBPII was administered at day 0, followed by MVA PvDBPII at 2 months.

Vaccinations in group 2 volunteers were interrupted by the COVID-19 pandemic and following a trial halt and amendment to the trial protocol, returning volunteers received a second dose of ChAd63 PvDBPII at 17 months, followed by MVA PvDBPII at 19 months. In the VAC079 trial, PvDBPII was administered at 0, 1 and 2 months. Vaccinations in group 1 volunteers were interrupted by the COVID-19 pandemic and after a trial halt, returning volunteers received their third vaccination at 14 months.

### Vaccine safety and immunogenicity

Following each vaccination, local and systemic adverse events (AEs) were self-reported by participants for 7 days using electronic diaries. Unsolicited and laboratory AEs were recorded for 28 days after each vaccination. Serious adverse events (SAEs) were recorded throughout the study period. Details on assessment of severity grading and causality of AEs are provided in the supplemental materials. Post-vaccination clinic reviews were conducted at days 1, 3, 7, 14 and 28 after each vaccination during which observations were taken, adverse events were elicited and blood was taken for hematology, biochemistry and immunology tests.

Total anti-PvDBPII IgG serum concentrations were assessed over time by ELISA using standardized methodology (6, 9). BIA which block the interaction of recombinant PvDBPII to DARC in vitro, were assessed in serum using an ELISA-based assay (6). In vitro GIA of 10 mg/mL purified total IgG was measured using a transgenic *P. knowlesi* parasite line expressing the PvDBP PvW1 allele (fig. S11), modified from a previous version expressing PvDBP SalI allele (33). The frequencies of IFN-γ^+^ PvDBPII-specific (SalI allele) CD4^+^ and CD8^+^ effector memory T cells were measured using flow cytometry.

### Controlled human malaria infection

Vaccinees underwent CHMI 2 to 4 weeks following their final vaccination and in parallel with unvaccinated infectivity controls in the VAC069 study. Blood-stage CHMI was initiated by intravenous injection of blood infected with the PvW1 clone of *P. vivax*, which originated from Thailand (8). PvW1 possesses a single copy of the PvDBP gene and its PvDBPII sequence is heterologous to SalI (8) (fig. S12). On the day of CHMI, aliquots of 0.5 mL cryopreserved PvW1 infected blood were thawed and each participant was challenged with a 1:10 dilution of one aliquot by intravenous injection into the forearm (8). One dose of the 1:10 diluted inoculum contained between 165 to 217 genome copies (gc) of *P. vivax* as quantified by qPCR, which will be an overestimate of the number of live viable parasites administered per volunteer.

From day 6 or 7 post-CHMI, participants were reviewed in clinic once to twice daily for symptoms of malaria and blood parasitemia was measured in real time by qPCR of the 18S ribosomal RNA gene (8). Laboratory staff carrying out qPCR were blinded to the volunteer study group and clinic staff were blinded to the qPCR result of volunteers until qPCR reached malaria diagnostic criteria. Volunteers were commenced on antimalarial treatment if they had substantial malaria symptoms and parasitemia ≥5,000 genome copies (gc)/mL; or if parasitemia reached ≥10,000 gc/mL irrespective of symptoms. Positive thick film microscopy was also included in the malaria diagnostic criteria in the CHMI trial in 2019 but was removed from later phases (Fig. 1). Treatment was with Riamet (60-hour course of artemether/lumefantrine) or Malarone (3-day course of atovaquone/proguanil hydrochloride). Outpatient review continued until completion of antimalarial treatment. Further follow-up visits took place at 2 and 3 months after the day of challenge for all volunteers, and 9 months after challenge in vaccinees only.

### Statistical analysis

For the primary efficacy analysis, pairwise comparison of qPCR-derived PMR was made between volunteers who received the same vaccine versus pooled data from all infectivity controls across three CHMIs using Mann-Whitney test. Post-hoc analysis comparing PMR between each vaccination regimen and infectivity controls was performed using Kruskal-Wallis test with Dunn’s multiple comparison post-test. The mean of three replicate qPCR results for each individual at each timepoint was used to model the PMR for each volunteer. Mean qPCR values that were below the lower limit of quantification (20 gc/mL) were excluded from further analyses. PMR was calculated from the slope of a linear model fitted to log_10_ transformed qPCR data (16). Exploratory analysis of parasite growth was conducted by calculating log_10_ cumulative parasitemia (LCP) for each individual up to the day on which the first volunteer reached malaria diagnostic criteria across all CHMIs.

Data were analyzed using GraphPad Prism version 8.3.1 for Windows (GraphPad Software Inc) and statistical tests are indicated in the text. Comparisons between more than two groups were performed using Kruskal-Wallis test with Dunn’s multiple comparison post-test. Correlations were assessed using Spearman’s rank correlation.

## Supplementary Material

Supplementary Materials and Methods

## Data Availability

All data associated with this study are in the paper or supplementary materials. Requests for datasets and materials should be addressed to the corresponding author, aside from requests for transgenic *P. knowlesi* lines, which are available from RWM under a material transfer agreement with the Francis Crick Institute and London School of Hygiene and Tropical Medicine. This research was funded in whole or in part by the Wellcome Trust [Grant number 212336/Z/18/Z], a cOAlition S organization. The author will make the Author Accepted Manuscript (AAM) version available under a CC BY public copyright license.

## One-Sentence Summary

The PvDBPII/Matrix-M vaccine, which targets the *Plasmodium vivax* Duffy-binding protein, reduced growth of malaria parasites in blood of humans.
