# Supplementary material for: Vaccination with Plasmodium vivax Duffy-binding protein inhibits parasite growth during controlled human malaria infection
Source: Sci Transl Med. Author manuscript; Available in PMC 2023 Sep 26. (PMC7615121; doi:10.1126/scitranslmed.adf1782)
Supplement: Supplementary Materials and Methods [file EMS188109-supplement-Supplementary_Materials_and_Methods.pdf]

Supplementary Materials and Methods

Trial approvals

The studies received ethical approval from UK National Health Service Research Ethics Services, (VAC069: Hampshire A Research Ethics Committee, Ref 18/SC/0577; VAC071: Oxford A Research Ethics Committee, Ref 19/SC/0193; VAC079: Oxford A Research Ethics Committee, Ref 19/SC/0330). The vaccine trials were approved by the UK Medicines and Healthcare products Regulatory Agency (VAC071: EudraCT 2019-000643-27; VAC079: EudraCT 2019-002872-14).

Trial inclusion and exclusion criteria

Inclusion and exclusion criteria for both vaccine trials (VAC071, VAC079) are listed below.

Inclusion criteria:

- Healthy adult aged 18 to 45 years.
- Red blood cells positive for the Duffy antigen/chemokine receptor (DARC).
- Normal serum levels of Glucose-6-phosphate dehydrogenase (G6PD).
- Able and willing (in the Investigator's opinion) to comply with all study requirements.
- Willing to allow the Investigators to discuss the volunteer's medical history with their General Practitioner.
- Women only: Must practice continuous effective contraception for the duration of the study
- Agreement to permanently refrain from blood donation.
- Written informed consent to participate in the trial.
- Reachable (24/7) by mobile phone during the period between controlled human malaria infection (CHMI) and completion of all antimalarial treatment.
- Willing to take a curative anti-malarial regimen following CHMI.
- Willing to reside in Oxford for the duration of the study, until antimalarials have been completed.
- Answer all questions on the informed consent quiz correctly.

Exclusion criteria:

- History of clinical malaria (any species).
- Travel to a clearly malaria endemic locality during the study period or within the preceding six months.
- Current or planned treatment with long-acting immune-modifying drugs at any time during the study period (e.g. infliximab).
- Chronic use of antibiotics with antimalarial effects (e.g. tetracyclines for dermatologic patients, trimethoprim-sulfamethoxazole for recurrent urinary tract infections, or others).
- Weight less than 50kg, as measured at the screening visit.
- Receipt of immunoglobulins within the three months prior to planned administration of the vaccine candidate.
- Receipt of blood products (e.g., blood transfusion) at any time in the past.

- Peripheral venous access unlikely to allow twice daily blood testing (as determined by the Investigator).
- Receipt of an investigational product in the 30 days preceding enrolment, or planned receipt during the study period.
- Receipt of any vaccine in the 30 days preceding enrollment, or planned receipt of any other vaccine within 30 days preceding or following each study vaccination, with the exception of licensed COVID-19 vaccines, which should not be received within 14 days before or 7 days after any study vaccination.
- Planned receipt of a COVID-19 vaccine between 2 weeks before the day of CHMI until completion of antimalarial treatment.
- Concurrent involvement in another clinical trial or planned involvement during the study period.
- Prior receipt of an investigational vaccine likely to impact on interpretation of the trial data or the *Plasmodium vivax* parasite as assessed by the Investigator.
- History of sickle cell anemia, sickle cell trait, thalassemia or thalassemia trait or any hematological condition that could affect susceptibility to malaria infection.
- Any confirmed or suspected immunosuppressive or immunodeficient state, including HIV infection; asplenia; recurrent, severe infections and chronic (more than 14 days); or immunosuppressant medication within the past 6 months (inhaled and topical steroids are allowed).
- History of allergic disease or reactions likely to be exacerbated by any component of the vaccine, such as egg products, Kathon, aminoglycosides.
- History of allergic disease or reactions likely to be exacerbated by malaria infection.
- History of clinically significant contact dermatitis.
- Any history of anaphylaxis in reaction to vaccinations.
- Pregnancy, lactation or intention to become pregnant during the study.
- Use of medications known to cause prolongation of the QT interval and existing contraindication to the use of Malarone.
- Use of medications known to have a potentially clinically significant interaction with Riamet and Malarone.
- Any clinical condition known to prolong the QT interval.
- History of cardiac arrhythmia, including clinically relevant bradycardia.
- Disturbances of electrolyte balance, e.g. hypokalemia or hypomagnesaemia.
- Family history of congenital QT prolongation or sudden death.
- Contraindications to the use of both of the proposed anti-malarial medications; Riamet Malarone.
- History of cancer (except basal cell carcinoma of the skin and cervical carcinoma in situ).
- History of serious psychiatric condition that may affect participation in the study.
- Any other serious chronic illness requiring hospital specialist supervision.
- Suspected or known current alcohol abuse as defined by an alcohol intake of greater than 25 standard UK units every week.
- Suspected or known injecting drug abuse in the 5 years preceding enrolment.
- Hepatitis B surface antigen (HBsAg) detected in serum.
- Seropositive for hepatitis C virus (antibodies to HCV) at screening, or (unless has taken part in a prior hepatitis C vaccine study with confirmed negative HCV antibodies prior to participation in that study, and negative HCV RNA polymerase chain reaction (PCR) at screening for this study).

- Positive family history in both 1st AND 2nd degree relatives < 50 years old for cardiac disease.
- Volunteers unable to be closely followed for social, geographic, or psychological reasons.
- Any clinically significant abnormal finding on biochemistry or hematology blood tests, urinalysis or clinical examination. In the event of abnormal test results, confirmatory repeat tests will be requested. Procedures for identifying laboratory values meeting exclusion criteria are shown in SOP VC027.
- Any other significant disease, disorder, or finding which may significantly increase the risk to the volunteer because of participation in the study, affect the ability of the volunteer to participate in the study or impair interpretation of the study data.
- Inability of the study team to contact the volunteer's GP to confirm medical history and safety to participate.

Peripheral Blood Mononuclear Cell (PBMC), plasma, and serum preparation

Blood samples were collected into lithium heparin-treated vacutainer blood collection systems. PBMC were frozen in fetal calf serum containing 10% dimethyl sulfoxide and stored in liquid nitrogen. Plasma samples were stored at -80 °C. For serum preparation, blood samples were collected into untreated vacutainers, incubated at room temperature and then the clotted blood was centrifuged for 5 min (750 *x g*). Serum was stored at -80 °C.

Anti-PvDBPII standardized enzyme-linked immunosorbent assay (ELISA)

ELISAs to quantify circulating PvDBPII-specific total IgG responses were performed using standardized methodology, similar to that previously described (*34*). Nunc MaxiSorp ELISA plates (Thermo Fisher) were coated overnight (≥16 h) at 4 °C with 50 µL per well of 2 µg/mL PvDBPII (SalI or PvW1 allele) protein (*34*). Plates were washed 6x with 0.05 % phosphate-buffered saline/Tween (PBS/T) and tapped dry. Plates were blocked for 1 h with 100 µL per well of Starting Block T20 (Thermo Fisher) at 20 °C. Test samples were diluted in blocking buffer (minimum dilution of 1:100), and 50 µL per well was added to the plate in triplicate. Reference serum (made from a pool of high-titer vaccinated donor serum) was diluted in blocking buffer in a three-fold dilution series to form a ten-point standard curve. Three independent dilutions of the reference serum were made to serve as internal controls. The standard curve and internal controls were added to the plate at 50 µL per well in duplicate. Plates were incubated for 2 h at 20 °C and then washed 6x with PBS/T and tapped dry. Goat anti-human IgG–alkaline phosphatase secondary antibody (Merck) was diluted 1:1000 in blocking buffer and 50 µL per well was added. Plates were incubated for 1 h at 20 °C. Plates were washed 6x with PBS/T and tapped dry. 100 µL per well of p-nitrophenyl phosphate alkaline phosphatase substrate (Thermo Fisher) was added, and plates were incubated for approximately 15 min at 20 °C. Optical density at 405 nm (OD_{405}) was measured using an ELx808 absorbance reader (BioTek) until

the internal control reached an OD_{405} of 1.0. The reciprocal of the internal control dilution giving an OD_{405} of 1.0 was used to assign an arbitrary unit (AU) value of the standard. Gen5 ELISA software v3.04 (BioTek) was used to convert the OD_{405} of test samples into AU by interpolating from the linear range of the standard curve fitted to a four-parameter logistic model. Any test samples with an OD_{405} below the linear range of the standard curve at the minimum dilution tested were assigned a minimum AU value of 5.0. These responses in AU are reported in µg/mL for the PvDBPII SalI allele following generation of a conversion factor by calibration-free concentration analysis (CFCA). In short, CFCA was performed using a Biacore X100 instrument, a Biotin CAP chip and X100 control and evaluation software (Cytiva). Purified mono-biotinylated antigen was produced for use in CFCA and chip was regenerated with manufacturer's supplied regeneration and CAP reagents and fresh antigen prior to each application of antibody. Serum samples, from a previous clinical trial (VAC051 (*34*)), with a range of PvDBP antibody responses were diluted and assessed for antigen-specific antibody binding and initial rates of antigen-specific binding at 5 µL/min and 100 µL/min measured and compared to permit measurement of concentration. The CFCA-measured PvDBP-specific antibody concentrations for each individual were analyzed by linear regression with corresponding total IgG ELISA AU data, where slope of the line was used to derive an AU-to-µg/mL conversion factor.

ELISA-based Binding Inhibition Assay

Samples were analyzed for binding inhibitory antibodies (BIA) at the Institut Pasteur, Paris, using previously reported methodology (*35*). Recombinant DARC-Fc (1 µg/mL) was coated on to a 96-well plate overnight at 4 °C in carbonate-bicarbonate buffer. Next day, the plate was blocked for 2 h at 37 °C using 2 % non-fat milk. Recombinant PvDBPII (SalI or PvW1 sequence) in a range of 0.8 to 25 ng/mL was used to generate a PvDBPII standard curve using a four-parameter logistic model. Serum samples were analyzed at dilutions of 1:10 to 1:2430. Each serum dilution was incubated with 25 ng/mL PvDBPII protein at 37 °C for 30 min. The reaction mixture was then added to DARC-Fc coated wells of an ELISA plate and incubated at 37 °C for 1 h. PvDBPII protein bound to recombinant DARC was probed with anti-PvDBPII polyclonal rabbit sera at 37 °C for 1 h and detected with anti-rabbit IgG HRP-conjugated secondary antibody at 37 °C for 1 h. The assay was developed using the two-component chromogenic substrate for peroxidase detection TMB (3,3′,5,5′-tetramethylbenzidine, Life Sciences) for 5 min and the reaction was stopped with phosphoric acid 1M (H_3PO_4). Absorbance was immediately measured at a wavelength of 450 nm. The amount of bound PvDBPII was estimated by converting OD values to protein concentrations using the PvDBPII standard curve. The interpolated protein concentration values were used to calculate percent binding for each serum sample dilution. The percent binding inhibition for each serum dilution was calculated as follows: % Binding Inhibition = 100 - % Binding. The plot of % Binding Inhibition versus serum

dilution was used to find the serum dilution at which 50% binding inhibition (IC_{50}) was achieved. Each assay was performed in duplicate and results from three independent replicates were used to determine average IC_{50}.

Plasmodium knowlesi parasites and growth inhibition activity (GIA) assay

It is not possible to culture blood-stage *P. vivax* long-term in vitro and therefore *P. vivax* parasites cannot be used at scale in GIA assays. Instead a transgenic *P. knowlesi* (a closely related simian malaria species) parasite line was generated, which is adapted to long-term in vitro culture in human red blood cells. We previously generated a transgenic *P. knowlesi* parasite line (*P. knowlesi* $PvDBP^{OR}\Delta\beta\Delta\gamma$), in which the PvDBP SalI allele transgene replaced the native PkDBPα gene, and the PkDBPβ and PkDBPγ genes were also knocked out (*36*). Here, we further modify this line to create a transgenic *P. knowlesi* line expressing the PvDBP PvW1 allele (**fig. S10**). A 20 bp guide sequence (CGA CAT CCT GAA GCA GGA AC) targeting the recodonized PvDBP (SalI) was identified and cloned into the PkCas9/sgRNA vector (pCas9/sg_PvDBPRII(SalI)^{OR}) as previously described (*36, 37*). A donor plasmid, pDonor_PvW1, was created by cloning a synthetic recodonized PvDBP PvW1 allele (GeneArt, Thermo Fisher) into the plasmid pDonor_PkDBPα^{OR}, using SpeI and NotI restriction sites. This created the final vector containing PvDBP PvW1 gene sequence flanked by 5' and 3' homology regions targeting the PkDBPα locus. The donor and guide were transfected into *P. knowlesi* $PvDBP^{OR}\Delta\beta\Delta\gamma$ using previously described methods (*36*). The resultant transfectants were cloned by limiting dilution and genotyped by PCR as described previously using diagnostic oligos for WT PvDBPRII^{OR} locus (ol186 fwd-CAC GAT TTG TGT ACT TAT AGA ATC AAT TTT TCC TT and ol189 rev-CGT TCT GGC CGT CGC CTG T) and for successful integration of PvDBP PvW1 allele (ol186 fwd and ol1799 rev-TCC CGT TCT TCC CAT CTC CGG T).

Samples were analyzed by the GIA Assay Reference Center, Laboratory of Malaria and Vector Research, National Institute of Allergy and Infectious Diseases, National Institutes of Health. The GIA assay methodology has been published elsewhere (*38*). In brief, 10 mg/mL purified total IgG (Protein G purified from serum) were mixed with about 1.5 % of trophozoite-rich parasites in a final volume of 40 μL in 96-well plates. After about 27 h of incubation, the relative parasitemia in each well was determined by parasite lactate dehydrogenase (pLDH) activity. Test IgG samples which showed greater than 10% GIA in the first assay were tested in two more independent assays, and median % GIA values from the three assays are reported.

Flow cytometry T cell assay

Flow cytometry was performed using frozen aliquots of PBMC from donors on day 0 (pre-vaccination) and 14 days and 28 days post-final vaccination with either VV-DBPII or PvDBPII/M-M. Cryopreserved PBMC were thawed and rested at 37°C before an 18 h 37°C stimulation in the dark with medium alone, 2.5 μg/peptide/mL of a PvDBPII 20mer peptide pool (Mimotopes) (**table S5**), or 1 μg/mL Staphylococcal enterotoxin B (SEB; S-4881, Sigma; positive control). Anti-CD28 (1 μg/mL; 16-0289-85, eBioscience, clone: CD28.2), anti-CD49d (1 μg/mL; 16-0499-85, eBioscience, clone: 9F10) and anti-CD107a-phycoerytrhin (PE)-cyanine (Cy) 5 (1/2550 dilution; 15-1079-42, eBioscience, clone: eBioH4A3) were included in the cell culture medium. Brefeldin A (00-4506-51, eBioscience) and monensin (00-4505-51, eBioscience) were added after 2 h. Following incubation, PBMC were stained and fixed with Cytofix/Cytoperm (554714, BD Biosciences). The following anti-human antibody and dye were used (20 minutes, room temperature, in the dark) prior to fixation: anti-CCR7-brilliant violet (BV) 711 (1/50 dilution; 353228, clone: G043H7, BioLegend); and Live/Dead Aqua (1/20 dilution; L34966, Invitrogen). The following anti-human antibodies and dyes were used (30 minutes, room temperature, in the dark) after fixation: anti-CD14-eFluor 450 (1/200 dilution; 48-0149-42, clone: 61D3), anti-CD19-eFluor 450 (1/200 dilution; 48-0199-42, clone: HIB19), anti-CD8a-allophycocyanine (APC)-eFluor 780 (1/10 dilution; 47-0088-42, clone: RPA-T8), anti-interferon (IFN)-γ-fluorescein isothiocyanate (FITC) (1/500 dilution; 11-7319-82, clone: 4S.B3), anti-tumor necrosis factor (TNF)-α-PE-Cy7 (1/2000 dilution; 25-7349-82, clone: MAb11), anti-CD3-Alexa Fluor 700 (1/100 dilution; 56-0038-82, clone: UCHT1) – all eBioscience; anti-CD4-peridinin chlorophyll protein (PerCP)-Cy5.5 (1/14 dilution; 300530, clone: RPA-T4), anti-interleukin (IL)-2-BV650 (1/50 dilution; 500334, clone: MQ1-17H12), anti-IL5-PE (1/40 dilution; 500904, clone: JES-39D10), anti-IL-13-APC (1/20 dilution; 501907, clone: JES10-5A2), anti-CD45RA-BV605 (1/2000 dilution; 304134, clone: HI100) – all BioLegend. Samples were acquired on a Fortessa flow cytometer using BD FACSDiva (both BD Biosciences) and data were analyzed in FlowJo (v10.8, Treestar).

Blood-stage inoculum preparation and CHMI

The PvW1 blood-stage inoculum was thawed and prepared under strict aseptic conditions as previously described (*39*). The required number of vials of the cryopreserved stabilate (each containing approximately 0.5 mL of red blood cells in 1 mL of Glycerolyte 57) were thawed in parallel in an area using solutions licensed for clinical use and single-use disposable consumables. A class II microbiological safety cabinet (MSC) was used to prepare the inoculum, which was fumigated with hydrogen peroxide and decontamination validated prior to use. To prepare the inoculum, 0.2 volume 12 % saline was added dropwise to the contents (about 1.5 mL) of each vial of thawed

infected blood. Each sample was left for 5 min, before an additional 10 volumes of 1.6 % saline was added dropwise prior to centrifugation for 4 min at 830 x *g*. Each supernatant was removed and 10 mL of 0.9 % saline was added dropwise. The cell pellets were pooled and washed twice in 0.9 % saline before a final resuspension into one 10 mL sample in 0.9 % saline. This 10 mL suspension was then divided into aliquots, equivalent to one tenth of one original cryovial. Each aliquot was made up to a total volume of 5 mL in 0.9 % saline in a sterile syringe for injection and transported to the clinic. For each challenge, one dose of the 1:10 diluted inoculum was quantified by quantitative polymerase chain reaction (qPCR) to be equivalent to between 165 to 217 genome copies of *P. vivax*. This will be an overestimate of the number of live viable parasites administered per volunteer because some parasites will be killed during the inoculum thawing and preparation process.

The reconstituted blood-stage inoculum (5 mL per syringe) was injected intravenously using an indwelling cannula, preceded and followed by a saline flush. The inoculum was administered to all volunteers within a maximum of 3 h 7 min from thawing of the inoculum. Volunteers were observed for 1 h following injection of the inoculum before discharge from the clinical facility. Following each CHMI, a leftover sample of the inoculum was cultured and shown to be negative for bacterial contamination.

Malaria parasite quantification by qPCR

qPCR was used to measure *P. vivax* parasitemia in volunteers' blood in real-time as previously described (*39*) using an assay that targets the 18S ribosomal RNA (rRNA) gene. DNA was extracted from 0.4 mL whole EDTA blood using a QIAsymphony SP robot, utilizing the Qiagen DSP Blood Midi Kit and the pre-loaded Blood 400 v6 extraction protocol, with a 100 µL elution in ATE buffer selected. Additionally, aliquots of baseline samples taken within 2 days pre-CHMI were spiked with a known concentration of positive control DNA to check there was no presence of PCR inhibitors in volunteers' blood prior to CHMI.

Following DNA extraction, a standard Taqman absolute quantitation was used against a standard curve to amplify a 183 bp PCR product from the multi-copy, highly conserved 18S ribosomal RNA genes of *Plasmodium spp.* qPCR used the following adapted oligonucleotide primers and probe (*40*): 18s forward primer 5'-AGG AAG TTT AAG GCA ACA ACA GGT-3', 18s reverse primer 5'-GCA ATA ATC TAT CCC CAT CAC GA-3' and shortened FAM labelled probe sequence 5'-TGA ACT AGG CTG CAC GCG-3', was run on an ABI StepOne Plus machine with v2.3 software. Default Universal qPCR (target FAM-NFQ-MGB) and quality control (QC) settings were used apart from the use of 40 cycles and 25 µL reaction volume.

This qPCR detects DNA from pan-*Plasmodium* species, but unlike the synchronous growth of *P. falciparum*, circulating *P. vivax*-infected red blood cells may contain up to 10 to 15 individual genomes (in blood-stage late trophozoites and schizonts) and can also include the presence of gametocytes. The qPCR score is therefore reported in genome copies/mL (gc/mL) as opposed to a quantity of parasites.

The standard curve was generated from dilution of a linearized plasmid encoding part of the *Plasmodium spp.* 18S ribosomal RNA gene and calibrated using known *P. falciparum* (Pf) spiked blood samples initially and then reference DNA extracted from whole blood from *P. vivax*-infected patient samples in Thailand where parasites had been quantified by microscopy (kindly provided by Mahidol University). Based upon earlier results obtained using dilution series of microscopically-counted cultured Pf parasites, a Pf-specific 18S rRNA Taqman qPCR showed a lower limit of quantification (LLQ, defined as % covariance [CV] <20%) of around 20 Pf parasites (p)/mL blood(*41*). Counted parasite dilution series results also suggested that the lower limit of probable detection (LLD, that is a probability of >50% of ≥1 positive result among three replicate qPCR reactions) is in the region of 5 p/mL, whereas samples at 1 p/mL are consistently negative (24/24 qPCR reactions). Positive results in this assay (even at very low detection) are thus essentially 100 % specific for genuine parasitemia, with positive results beneath the LLQ likely to signify parasitemia in the range of 2 to 20 p/mL. Similar sensitivity in terms of genome copy detection was observed when using the pan-*Plasmodium* qPCR described above and the diluted *P. vivax*-infected patient blood test samples from Thailand. As noted, these samples had microscopically mixed life stages with varying copies of the 18S rRNA gene and thus the assay readout is reported in terms of gc/mL. Based on this and the above experiments, 20 gc/mL was set as the lower limit of detection to meet positive reporting criteria, but all raw data are shown in the Results.

For QC purposes, qPCR samples were re-tested if replicates included a mixture of positive and negative (in terms of amplification) results with one or more positive results > 100 gc/mL or if the % CV of any results were high outliers. All 'passed' data following the quality control steps above, including any 0 values, were used to generate the final mean qPCR result for each time-point.

Thick blood film microscopy

Collection of blood, preparation of thick films and slide reading were performed according to Jenner Institute Standard Operating Procedure (SOP) ML009. Slides were prepared using Field's stain A and then Field's stain B. 200 fields at high power (1000x) were read. Visualization of two or more parasites in 200 high power fields constituted a positive result. For internal quality control, all slides were read separately by two experienced microscopists, with a third read if results were discordant (one negative and one positive report).

Modelling of parasite multiplication rate

A qPCR-derived parasite multiplication rate (PMR) was modelled based on previously described methodology with modifications (*39, 41*). The arithmetic mean of three replicate qPCR results obtained for each individual at each time-point was used for model-fitting. Negative replicates and any qPCR data points below 20 gc/mL, based upon the mean of the three replicates, were removed prior to model-fitting. Data from timepoints in CHMIs conducted in September 2019 and May 2021, which would not have been available if using the visit schedule for the final CHMI in October 2021 (VAC069D /VAC071B /VAC079B), were also removed prior to model-fitting. The time interval between the morning and evening bleeds used for qPCR monitoring was set as 0.37 days. PMR per 48 h was then calculated using a linear model fitted to $\log_{10}$-transformed qPCR data.

Analysis of $\log_{10}$ cumulative parasitemia during CHMI

The arithmetic mean of three replicate qPCR results obtained for each individual at each time-point up until day C+14, when the first volunteer reached malaria diagnostic criteria across all CHMIs, was used for analysis. As per PMR modelling, negative replicates and any qPCR data points below 20 gc/mL, based upon the mean of the three replicates, were removed prior to analysis, as well as removal of data from timepoints in CHMIs conducted in September 2019 and May 2021, which would not have been available if using the visit schedule for the final CHMI in October 2021. Log_{10} cumulative parasitemia was then calculated from area under the curve analysis of $\log_{10}$-transformed qPCR data, where a peak was defined as any positive value above baseline.

Supplementary Figures

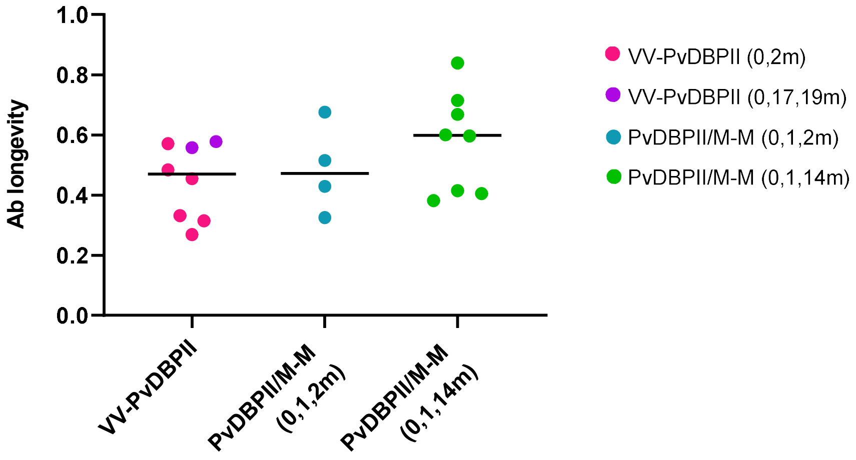

Figure S1. Antibody (Ab) longevity post-final vaccination.

For each individual, the area under the curve was calculated from the time of their peak anti-PvDBPII (SalI) total IgG response until the final timepoint available and divided by their peak antibody titer and duration over which the area under the curve was calculated in order to estimate the antibody longevity. Comparisons between groups with Kruskal-Wallis test were not statistically significant. m, month. Group medians are shown. Colored symbols indicate vaccination regimens.

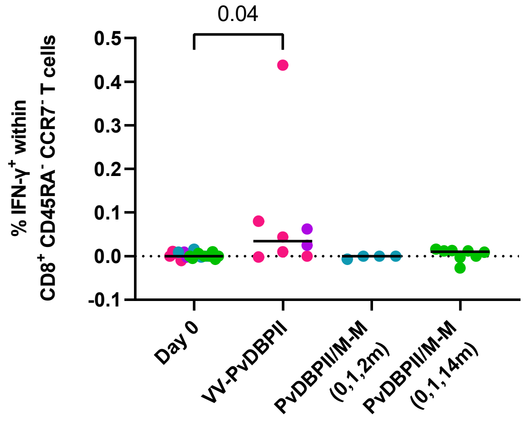

Figure S2. PvDBPII-specific CD8$^+$ T cell responses 14 days post-final vaccination.

Percentage of IFN-γ$^+$ cells within CD8$^+$ CD45RA$^-$ CCR7$^-$ effector memory T cells at 14 days post-final vaccination following PBMC stimulation with a pool of PvDBPII (SalI) peptides. The frequency of IFN-γ$^+$ cells in sample-matched unstimulated wells was subtracted to control for non-specific activation. Baseline responses (Day 0) are shown for all volunteers. *p* value as calculated by Kruskal-Wallis test with Dunn's multiple comparison post-test. Group medians are shown. Colored symbols indicate vaccination regimens as in fig. S1.

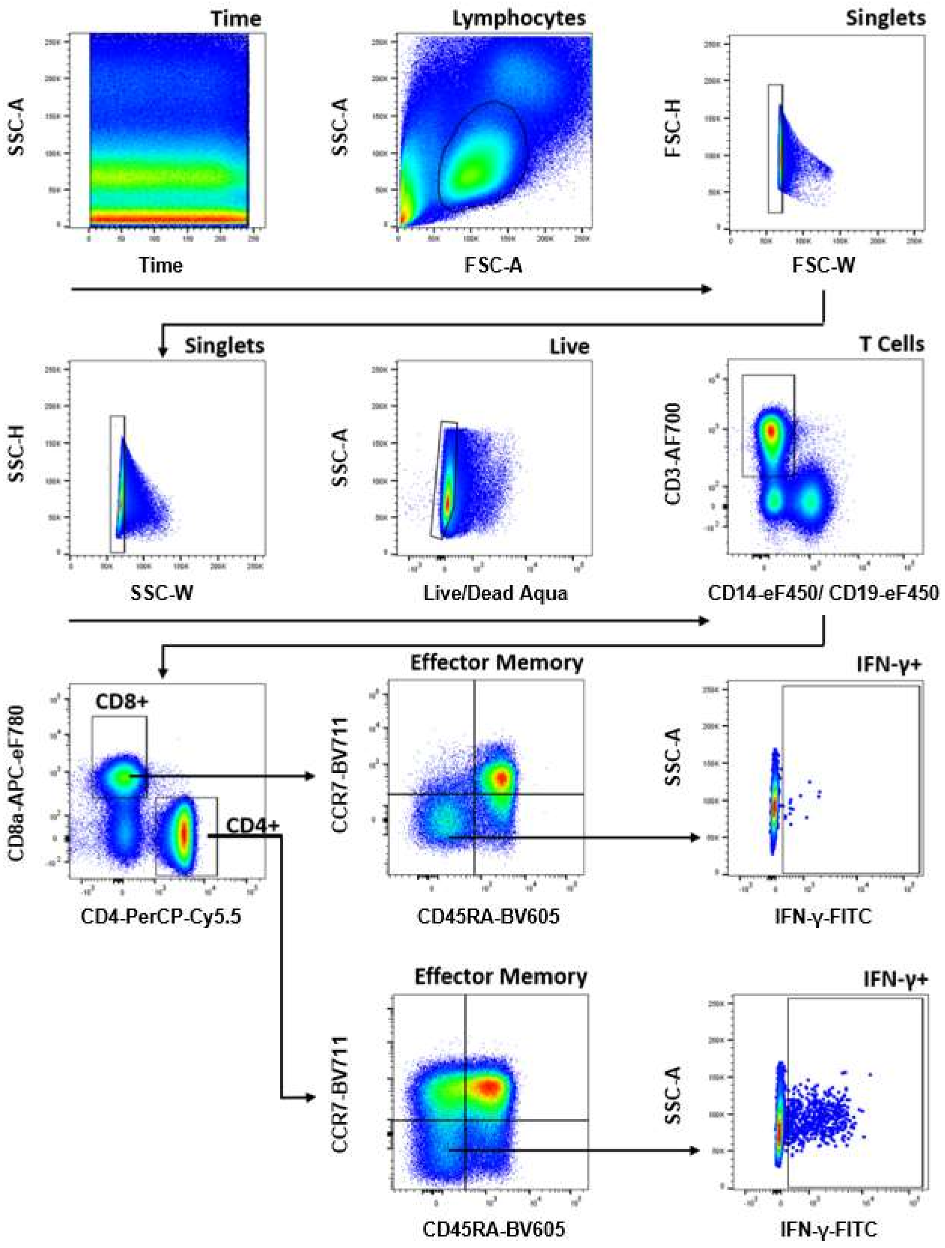

Figure S3. Flow cytometry gating strategy.

Gating strategy for definition of live singlet CD4$^+$ and CD8$^+$ effector memory T cells, and for gating of IFN-γ^+ cells within the live singlet CD4$^+$ and CD8$^+$ effector memory T cell populations. SSC, side scatter; FSC, forward scatter; A, area; H, height; W, width; eF, eFluor; AF, Alexa Fluor.

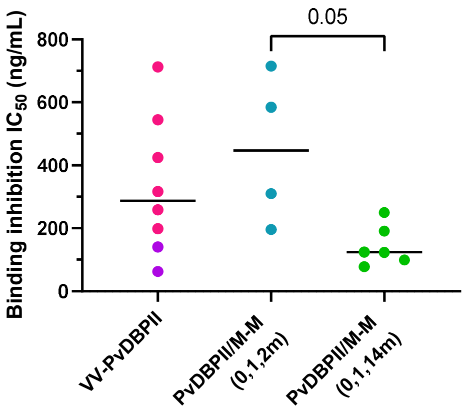

Figure S4. DARC-PvDBPII binding inhibition.

The concentration of anti–PvDBPII total IgG that is required to achieve 50% binding inhibition (IC_{50}) was calculated for each individual by dividing their serum anti-PvDBPII (SalI) total IgG concentration by the dilution factor of serum required to inhibit binding of DARC to PvDBPII (SalI) by 50%. Group medians are shown. *p* value as calculated by Kruskal-Wallis test with Dunn's multiple comparison post-test. Colored symbols indicate vaccination regimens as in fig. S1.

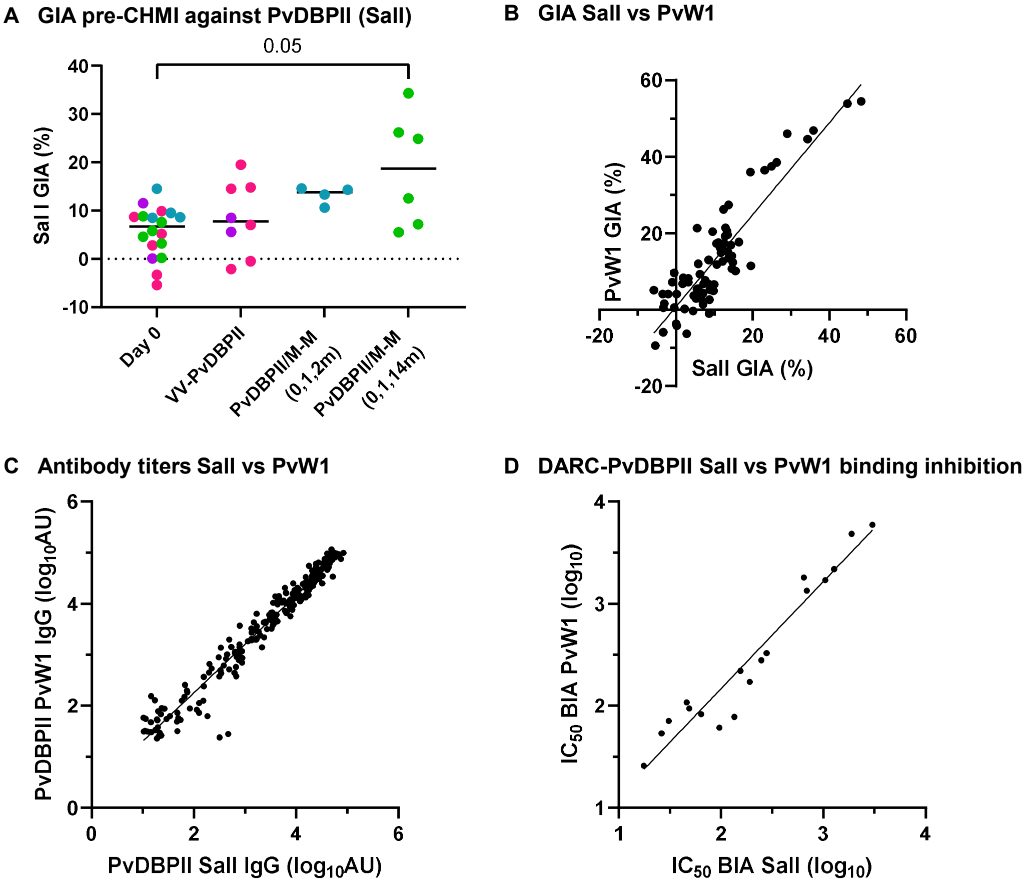

E

	Growth inhibition	Antibody titers	Binding inhibition
**Slope**	1.20	0.95	1.05
**95% CI**	1.07 to 1.34	0.93 to 0.98	0.90 to 1.20
**R^2**	0.81	0.95	0.93
**n**	75	230	18

Figure S5. Analysis of anti-PvDBPII SalI versus PvW1 GIA, ELISA and BIA responses.

(**A**) Percentage in vitro growth inhibition activity (GIA) of 10 mg/mL total IgG, taken pre-CHMI, against *P. knowlesi* expressing the PvDBP SalI allele. Baseline responses (Day 0) are shown for all volunteers. *p* values were calculated by Kruskal-Wallis test with Dunn's multiple comparison post-test. Horizontal lines indicate group medians. Colored symbols indicate vaccination regimens as in fig. S1. (**B**) Data from all volunteers and timepoints at which GIA of 10 mg/mL total IgG against both *P. knowlesi* expressing the PvDBP PvW1 allele and *P. knowlesi* expressing the PvDBP SalI allele are shown with linear regression line. (**C**) ELISA data from all volunteers and timepoints at which anti-PvDBPII total IgG responses against SalI and PvW1 alleles were assessed in serum. Responses to PvDBPII are reported in log_{10} arbitrary units (AU) with linear regression line. (**D**) Data from all volunteers and timepoints at which BIA in serum were assessed by inhibition of recombinant DARC-

PvDBPII binding against SalI and PvW1 alleles. Log_{10} dilution factors of individual serum required to inhibit binding of DARC to PvDBPII by 50% (IC_{50}) are reported with linear regression line. (**E**) Table summarizing parameters of linear regression of correlations shown in panels B, C and D. Slope, 95% confidence interval (CI) of slope, R squared and number of observations for each linear regression are shown.

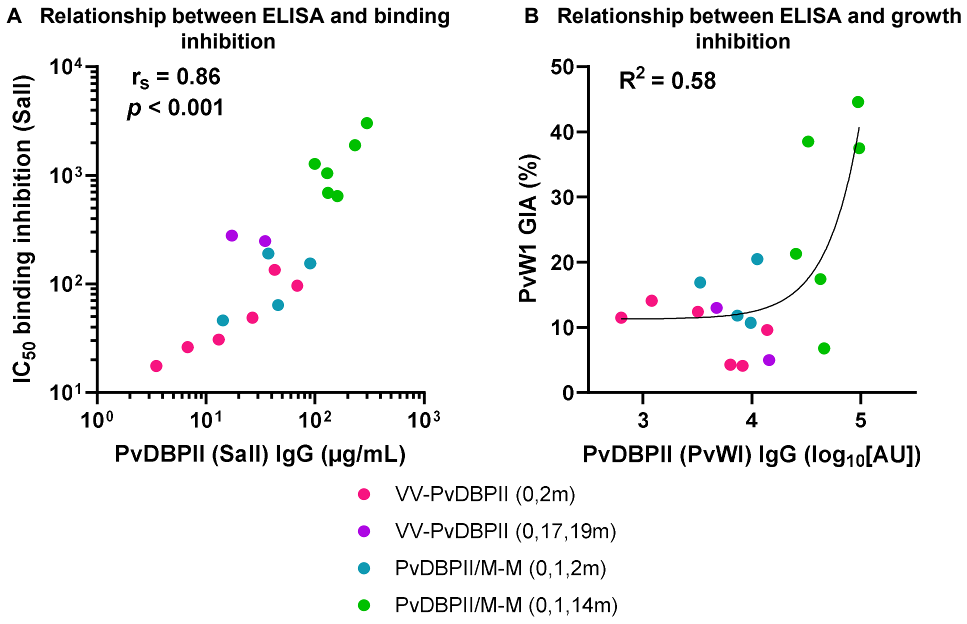

Figure S6. Relationships between measures of anti-PvDBPII antibody responses pre-CHMI.

(**A**) Correlation analysis between anti-PvDBPII (SalI) total IgG serum responses measured pre-CHMI by ELISA versus BIA measured at the same timepoint by IC_{50} values. Spearman's rank correlation coefficient and *p* value are shown, n=18. (**B**) Relationship between pre-CHMI *P. knowlesi* (PvW1) GIA assay data and anti-PvDBPII (PvWI) IgG response measured by ELISA in the purified serum IgG used in the assay. A non-linear regression curve is shown for all samples combined (solid line, n=18).

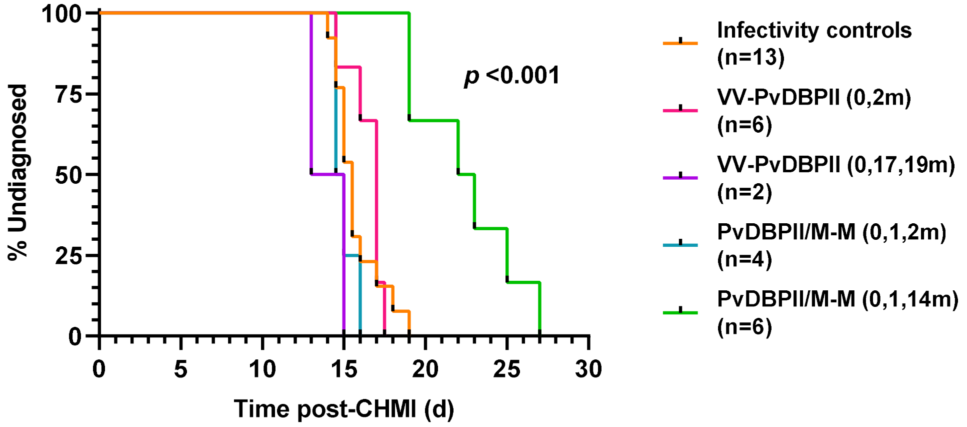

Figure S7. Kaplan-Meier plot of time to malaria diagnosis.

Median time to diagnosis was 15.5 days for controls and 22.5 days for PvDBPII/M-M delayed dosing regimen. Pairwise comparison with log-rank test between controls versus vaccine regimen groups was only significant for PvDBPII/M-M (0,1,14m) group (p <0.001).

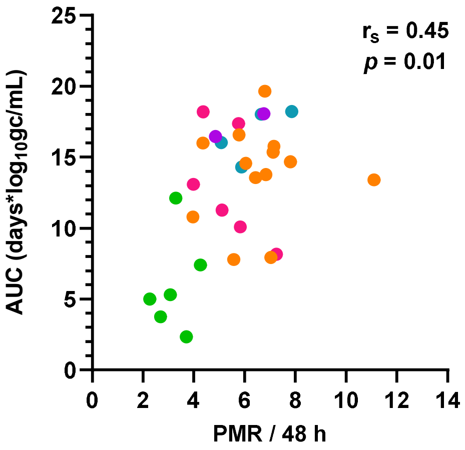

Figure S8. Relationship between in vivo parasite growth as measured by parasite multiplication rate versus $\log_{10}$ cumulative parasitemia.

Spearman's rank correlation coefficient and *p* value are shown, n=31. AUC, area under the curve. Colors indicate groups as in fig. S7.

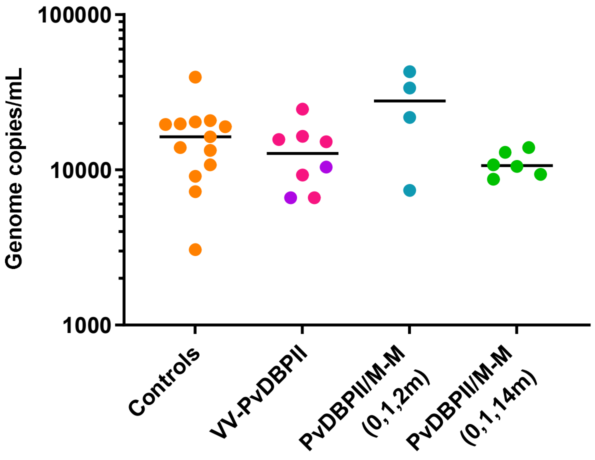

Figure S9. Malaria qPCR at diagnosis.

Parasitemia was measured by qPCR in gc/mL just prior to commencing anti-malarial treatment. Individual data and median values are shown. No significant differences were observed between the groups as measured by Kruskal-Wallis test with Dunn's multiple comparison post-test. Colors indicate groups as in fig. S7.

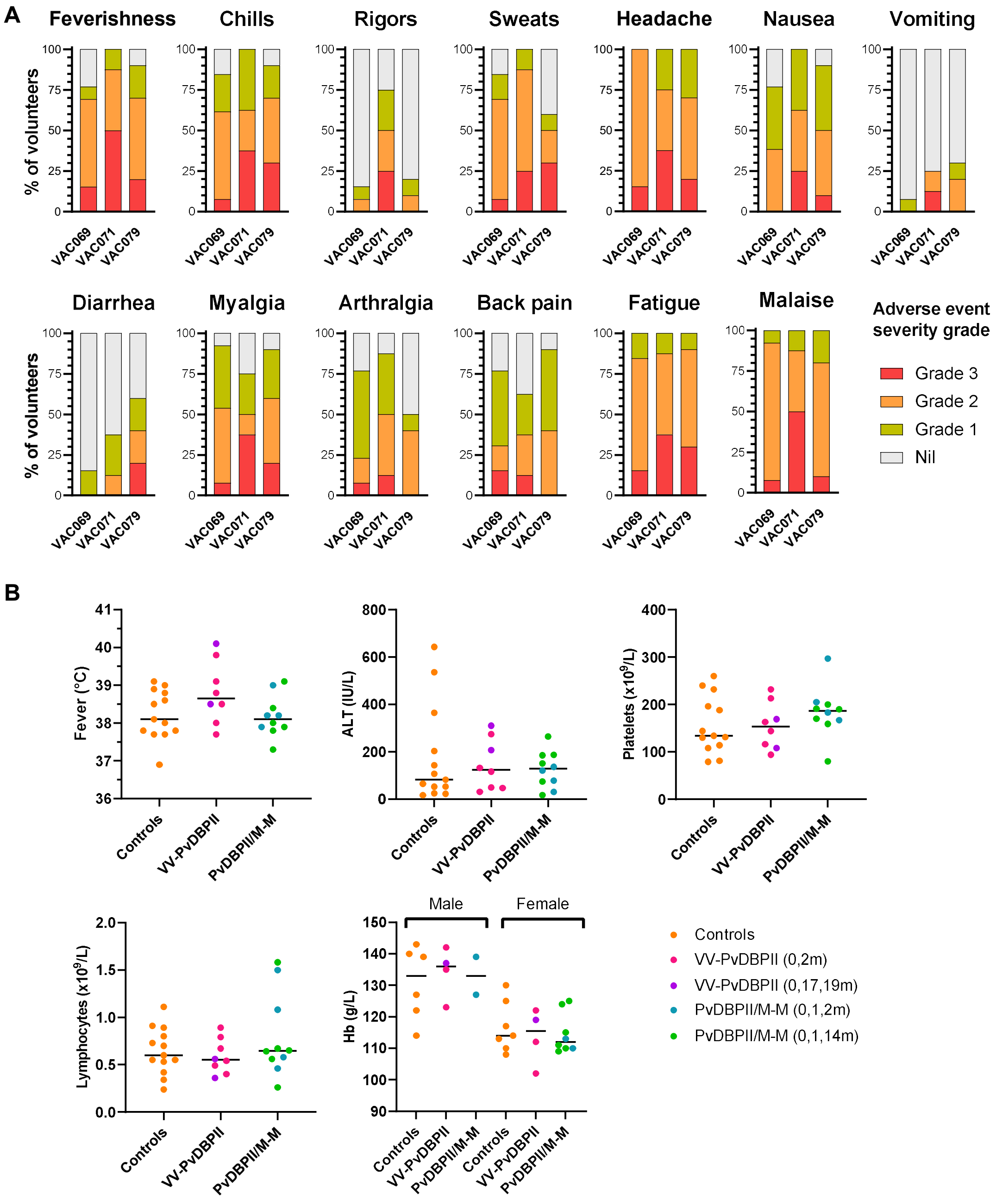

Figure S10. Clinical and laboratory adverse events during CHMI.

(**A**) Percentage of volunteers reporting solicited adverse events (AE) relating to malaria symptoms during CHMI. Maximum severity of each solicited AE reported by an individual is shown for control volunteers (VAC069, n=13), VV-PvDBPII vaccinees (VAC071, n=8) and PvDBPII/M-M vaccinees (VAC079, n=10). (**B**) Maximum recorded temperature, highest alanine transferase (ALT), lowest platelets, lowest lymphocytes and lowest hemoglobin (Hb) during CHMI is shown with medians. IU, international units. Horizontal lines represent medians.

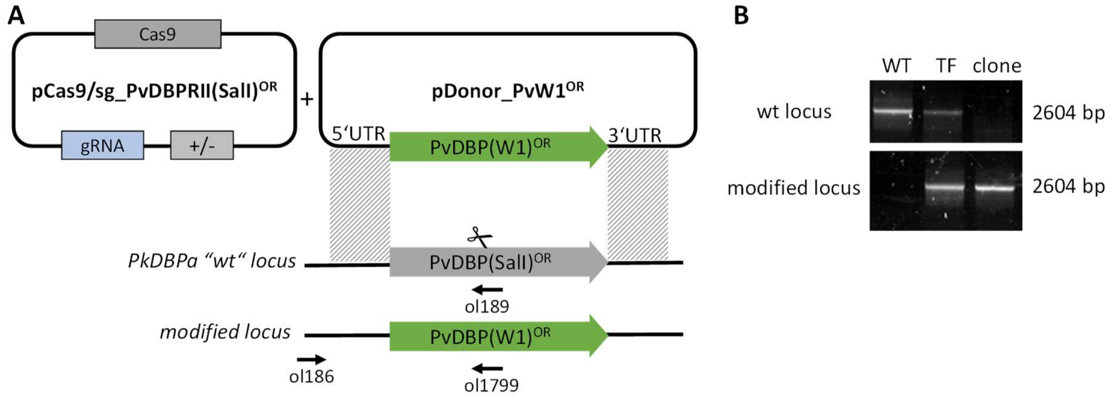

Figure S11. Design and genotypic analysis of *P. knowlesi* PvDBP PvW1 orthologue replacement line.

(**A**) Schematic detailing the approach to replace the coding sequence of PvDBP SalI allele with that of the PvDBP PvW1 allele within the previously generated transgenic *P. knowlesi* PvDBP^{OR}ΔβΔγ ("wild type", WT) strain. The pCas9/sg_PvDBPRII(SalI)^{OR} plasmid was transfected alongside the pDonor_PvW1^{OR}, to create a double strand break within the PvDBP SalI coding sequence and replace this with the PvDBP PvW1 allele by homologous recombination. Arrows indicate positions of diagnostic primers used for genotypic analysis of transfectants. (**B**) Parasites were analyzed by diagnostic PCR. Primer pairs were used to specifically detect wt locus (ol186 + ol189) and the modified locus (ol186 +ol1799) within i) the parental *P. knowlesi* PvDBP^{OR}ΔβΔγ (WT) strain; ii) bulk culture of transfectants (TF); and iii) a clonal transfectant (clone). The clonal *P. knowlesi* PvDBP^{OR} PvW1 transgenic parasite line was shown to only contain parasites with the PvW1 allele modified locus.

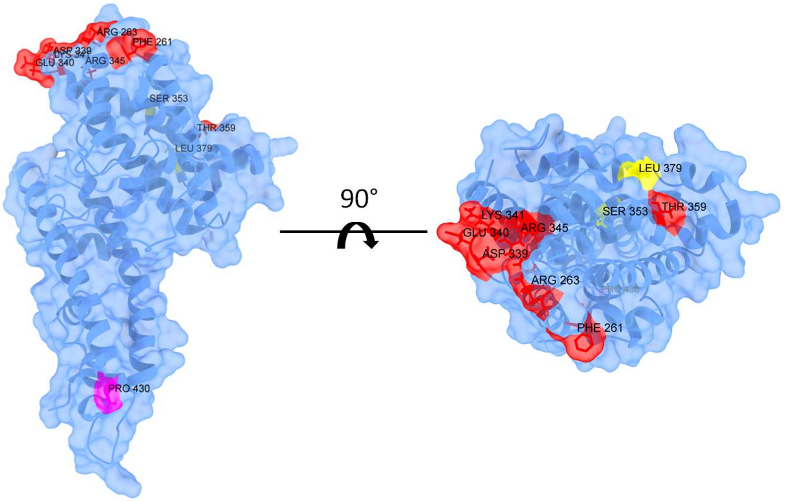

Amino acid position	261	263	339	340	341	345	353	359	379	430
**PvW1**	L	S	G	K	N	H	T	R	I	L
**PvSalI**	F	R	D	E	K	R	S	T	L	-

Figure S12. PvW1 polymorphisms mapped to the structure of PvDBPII SalI protein.

PvDBPII SalI strain (vaccine sequence) X-ray crystallography structure (PDB: 4NUU) annotated with sites of polymorphisms found in the PvWI strain (used for blood-stage CHMI) (*39*). Sites of non-conservative substitutions are shown in red, sites of conservative substitutions are shown in yellow, and sites of insertions are shown in pink. The table lists the amino acid polymorphisms within PvDBPII between SalI and PvW1 strains. The PvW1 sequence has a leucine insertion between positions 429 and 430 in the SalI sequence.

Supplementary Tables

Table S1. Baseline demographics of study participants. VV-PvDBPII indicates the viral-vectored vaccine; PvDBPII/M-M indicates the protein in Matrix-M adjuvant vaccine; CHMI indicates those volunteers who underwent controlled human malaria infection. *Samples from volunteers who were not heterozygous on Duffy blood group antigen (Fy) serophenotyping were also sent for Duffy blood group antigen genotyping. Only one volunteer (in the control group) with $Fya^{-}Fyb^{+}$ serophenotype had the Duffy blood group antigen genotype $FY*B/FY*B^{ES}$, whereby the erythrocyte silent (ES) allele has a mutation that prevents Fyb antigen expression in red blood cells.

		CHMI Controls	VV-PvDBPII		PvDBPII/M-M	
			All vaccinees	CHMI	All vaccinees	CHMI
No. of participants		13	16	8	16	10
Sex no.	Female	7	7	4	12	8
Age - median (range)		26 (21, 48)	25.5 (20, 44)	29 (21, 41)	28.5 (19, 44)	37 (21, 44)
Ethnicity no.	White	10	13	8	15	10
	Asian	1	2	0	0	0
	Arab	1	1	0	0	0
	Mixed	1	0	0	1	0
Duffy phenotype no.	$Fya^{+}Fyb^{-}$	3		2		1
	$Fya^{-}Fyb^{+}$	2*		4		3
	$Fya^{+}Fyb^{+}$	8		2		6

Table S2. Laboratory abnormalities following vaccinations. Number of episodes of laboratory abnormalities within 28 days following vaccinations with chimpanzee adenovirus 63 (ChAd63) PvDBPII, modified vaccinia virus Ankara (MVA) PvDBPII, or PvDBPII/Matrix-M. Maximal grade of laboratory abnormality, deemed at least possibly related to vaccination, is reported. V1 indicates first vaccination, V2 indicates second vaccination, and V3 indicates third vaccination.

Laboratory abnormality	Number of episodes					
	ChAd63 PvDBPII		MVA PvDBPII	PvDBPII/M-M		
	V1 (n=16)	V2 (n=2)	V1 (n=8)	V1 (n=16)	V2 (n=15)	V3 (n=12)
Leukopenia						
Grade 1	2	0	0	0	0	0
Lymphopenia						
Grade 1	5	1	0	0	4	2
Grade 2	6	0	2	1	1	2
Neutropenia						
Grade 1	3	0	0	0	0	1
Grade 2	2	0	0	0	0	0
Eosinophilia						
Grade 1	1	0	0	0	0	0
Hypokalemia						
Grade 1	3	1	0	0	0	0
Grade 3	0	1	0	0	0	0
Hyperbilirubinemia						
Grade 1	1	0	0	0	0	0
Anemia						
Grade 1	0	0	0	0	0	1
Thrombocytopenia						
Grade 1	0	0	0	1	0	0

Table S3. Unsolicited adverse events (AE) following ChAd63 or MVA PvDBPII vaccination. Unsolicited AEs occurring within 28 days of vaccination and deemed at least possibly related to ChAd63 or MVA PvDBPII vaccination. Number of episodes of unsolicited AE are listed by vaccine and MEDDRA System Organ Class and Preferred Term. Unsolicited AEs were of maximal grade 2 severity.

Unsolicited AE	Number of episodes of AE	
	ChAd63 PvDBPII	MVA PvDBPII
Gastrointestinal disorders		
Diarrhea	1	0
Abdominal pain upper	1	0
Vomiting	1	0
Dry mouth	1	0
Respiratory, thoracic and mediastinal disorders		
Rhinitis	1	1
Oropharyngeal pain	1	1
Cough	0	1
Skin and subcutaneous tissue disorders		
Rash	1	0
Musculoskeletal and connective tissue disorders		
Neck pain	1	0
Pain in extremity	1	0
Reproductive system and breast disorders		
Dysmenorrhea	2	0
Eye disorders		
Dry eye	1	0
General disorders and administration site conditions		
Chest pain	1	0
Nervous system disorders		
Paresthesia	0	1
Metabolism and nutrition disorders		
Decreased appetite	0	1

Table S4. Unsolicited AEs following PvDBPII/M-M vaccination. Unsolicited AEs occurring within 28 days of vaccination and deemed at least possibly related to PvDBPII/M-M vaccination. Number of episodes of unsolicited AE following the first, second, and third vaccination are listed by MEDDRA System Organ Class and Preferred Term. Unsolicited AEs were of maximal grade 2 severity.

Unsolicited AE	Number of episodes of AE with PvDBPII/M-M			
	V1 (n=16)	V2 (n=15)	V3 (n=12)	Total
Gastrointestinal disorders				
Diarrhea	0	1	0	1
General disorders and administration site conditions				
Administration site induration	1	0	0	1
Chest discomfort	1	0	0	1
Injection site pruritus	2	0	0	2
Injection site swelling	1	0	0	1
Swelling	1	0	0	1
Infections and infestations				
Rhinitis	1	0	0	1
Musculoskeletal and connective tissue disorders				
Back pain	2	1	0	3
Pain in extremity	2	1	0	3
Nervous system disorders				
Dizziness	1	0	0	1
Headache	0	2	1	3
Migraine	1	0	0	1
Hypoesthesia	0	1	0	1
Paresthesia	2	0	0	2
Somnolence	0	1	0	1
Taste disorder	1	0	0	1
Psychiatric disorders				
Euphoric mood	1	0	0	1
Insomnia	1	1	1	3
Tearfulness	1	0	0	1
Reproductive system and breast disorders				
Dysmenorrhea	0	1	0	1
Respiratory, thoracic and mediastinal disorders				
Nasal congestion	1	0	0	1
Oropharyngeal pain	0	1	0	1

Throat irritation	1	1	0	2

Table S5. PvDBPII peptides used for T cell stimulation. The PvDBPII SalI amino acid sequence was used to design 20mer peptides overlapping by 12 amino acids and these were synthesized by Mimotopes. Each stock was reconstituted to 50 mg/mL in dimethyl sulfoxide. A 200 µg/peptide/mL working stock of PvDBPII peptides was prepared by adding an equal amount of each peptide to cell culture medium for a final total peptide concentration of 8 mg/mL.

Peptide Number	N-terminus	Amino Acid Sequence	C-terminus
1	H-	DHKKTISSAIINHAFLQNTVGSG(261)	-NH2
2	Biotin-	SGSGAIINHAFLQNTVMKNCNYKR	-NH2
3	Biotin-	SGSGQNTVMKNCNYKRKRRERDWD	-NH2
4	Biotin-	SGSGNYKRKRRERDWDCNTKKDVC	-NH2
5	Biotin-	SGSGRDWDCNTKKDVCIPDRRYQL	-NH2
6	Biotin-	SGSGKDVCIPDRRYQLCMKELTNL	-NH2
7	Biotin-	SGSGRYQLCMKELTNLVNNTDTNF	-NH2
8	Biotin-	SGSGLTNLVNNTDTNFHRDITFRK	-NH2
9	Biotin-	SGSGDTNFHRDITFRKLYLKRKLI	-NH2
10	Biotin-	SGSGTFRKLYLKRKLIYDAAVEGD	-NH2
11	Biotin-	SGSGRKLIYDAAVEGDLLLKLNNY	-NH2
12	Biotin-	SGSGVEGDLLLKLNNYRYNKDFCK	-NH2
13	Biotin-	SGSGLNNYRYNKDFCKDIRWSLGD	-NH2
14	Biotin-	SGSGDFCKDIRWSLGDFGDIIMGT	-NH2
15	Biotin-	SGSGSLGDFGDIIMGTDMEGIGYS	-NH2
16	Biotin-	SGSGIMGTDMEGIGYSKVVENNLR	-NH2
17	Biotin-	SGSGIGYSKVVENNLRSIFGTDEK	-NH2
18	Biotin-	SGSGNNLRSIFGTDEKAQQRRKQW	-NH2
19	Biotin-	SGSGTDEKAQQRRKQWWNESKAQI	-NH2
20	Biotin-	SGSGRKQWWNESKAQIWTAMMYSV	-NH2
21	Biotin-	SGSGKAQIWTAMMYSVKKRLKGNF	-NH2
22	Biotin-	SGSGMYSVKKRLKGNFIWICKLNV	-NH2
23	Biotin-	SGSGKGNFIWICKLNVAVNIEPQI	-NH2
24	Biotin-	SGSGKLNVAVNIEPQIYRWIREWG	-NH2
25	Biotin-	SGSGEPQIYRWIREWGRDYVSELP	-NH2
26	Biotin-	SGSGREWGRDYVSELPTEVQKLKE	-NH2
27	Biotin-	SGSGSELPTEVQKLKEKCDGKINY	-NH2
28	Biotin-	SGSGKLKEKCDGKINYTDKKVCKV	-NH2
29	Biotin-	SGSGKINYTDKKVCKVPPCQNACK	-NH2
30	Biotin-	SGSGVCKVPPCQNACKSYDQWITR	-NH2
31	Biotin-	SGSGNACKSYDQWITRKKNQWDVL	-NH2

32	Biotin-	SGSGWITRKKNQWDVLSNKFISVK	-NH2
33	Biotin-	SGSGWDVLSNKFISVKNAEKVQTA	-NH2
34	Biotin-	SGSGISVKNAEKVQTAGIVTPYDI	-NH2
35	Biotin-	SGSGVQTAGIVTPYDILKQELDEF	-NH2
36	Biotin-	SGSGPYDILKQELDEFNEVAFENE	-NH2
37	Biotin-	SGSGLDEFNEVAFENEINKRDGAY	-NH2
38	Biotin-	SGSGFENEINKRDGAYIELCVCSV	-NH2
39	Biotin-	SGSGDGAYIELCVCSVEEAKKNTQ	-NH2
40	Biotin-	SGSGIELCVCSVEEAKKNTQEVVT	-OH

Table S6. Malaria qPCR data (gc/mL) for CHMI in September 2019. Malaria qPCR data used in PMR modelling are shown. The top row represents day (D) of follow-up visit post blood-stage CHMI. DoD indicates the timepoint at which malaria diagnostic criteria were reached. VAC069 Group 6, infectivity controls; VAC071 Group 1, VV-PvDBPII given at 0, 2 months. Treatment in some volunteers was started half a day after reaching malaria diagnostic criteria. qPCR data shown for samples taken prior to starting treatment. qPCR negative values for all three triplicate readings in the assay are indicated by ‘N’. Squares highlighted in gray indicate negative or < 20 gc/mL which is below minimum positive reporting criteria and these datapoints were removed for PMR modelling. Datapoints which would not have been taken during CHMI in October 2021 due to changes in protocol were removed for PMR modelling and are not shown. Blacked out boxes indicate the timepoints after a volunteer commenced antimalarial treatment.

Trial	Group	DoD	D7	D8	D9	D10	D11	D11.5	D12	D12.5	D13	D13.5	D14	D14.5	D15	D15.5	D16	D16.5	D17	D17.5
VAC069	6	15.5	N	N	45	104	209		270		1379	1391	2244	3670	8921	9574	16345			
VAC069	6	14.5	N	9	72	N	265		778		3365	2835	4283	17392	19589					
VAC071	1	17	11	9	N	16	54		198		608		792		3098	3281	3694	4831	16135	15663
VAC071	1	17	N	N	26	13	80		94		362		652		2768	2751	4192	3832	15176	
VAC071	1	17.5	N	27	31	44	74		123		527		1285	889	2745	2295	2465	2914	9588	9263

Table S7. Malaria qPCR data (gc/mL) for CHMI in May 2021. Malaria qPCR data used in PMR modelling are shown. Top row represents day (D) of follow-up visit post blood-stage CHMI. DoD indicates the timepoint at which malaria diagnostic criteria were reached. VAC069 Group 9, infectivity controls; VAC079 Group 1, PvDBPII/M-M given at 0, 1, 14 months. Treatment in some volunteers was started half a day after reaching malaria diagnostic criteria. qPCR data shown for samples taken prior to starting treatment. qPCR negative values for all three triplicate readings in the assay are indicated by 'N'. Squares highlighted in gray indicate negative or < 20 gc/mL which is below minimum positive reporting criteria and these datapoints were removed for PMR modelling. Datapoints which would not have been taken during CHMI in October 2021 due to changes in protocol were removed for PMR modelling and are not shown. Blacked out boxes indicate the timepoints after a volunteer commenced antimalarial treatment.

Trial	Group	DoD	D7	D8	D9	D10	D11	D11.5	D12	D12.5	D13	D13.5	D14	D14.5	D15	D15.5	D16	D16.5	D17	D17.5	D18
VAC069	9	15.5	**N**	**N**	**74**	**220**	**316**		**408**		**1678**	**1315**	**3625**	**6183**	**8674**	**9095**					
VAC069	9	18	**10**	**N**	**8**	**N**	**125**		**88**		**291**		**814**	**1252**	**1652**	**1805**	**2152**	**6550**	**7261**	**6161**	**12050**
VAC069	9	19	**N**	**N**	**26**	**37**	**98**		**62**		**268**		**298**		**1111**	**1262**	**1617**	**2121**	**6005**	**6395**	**4758**
VAC069	9	14	**32**	**35**	**108**	**182**	**1059**	**748**	**1121**	**2572**	**8025**	**7805**	**14766**	**18983**							
VAC069	9	16	**11**	**N**	**47**	**74**	**311**		**298**		**1669**	**1544**	**3316**	**6509**	**8709**	**9292**	**15568**	**39496**			
VAC069	9	14.5	**N**	**N**	**50**	**96**	**370**		**754**		**2641**	**2335**	**4847**	**12160**	**7238**						
VAC069	9	15	**N**	**N**	**113**	**183**	**485**		**556**		**3001**	**2476**	**5926**	**7904**	**16068**	**20776**					
VAC079	1	19	**6**	**21**	**17**	**31**	**79**		**73**		**316**		**379**		**798**		**1038**	**1474**	**3570**	**3555**	**3665**
VAC079	1	19	**N**	**N**	**18**	**15**	**77**		**135**		**155**		**442**		**719**		**1101**	**1578**	**3775**	**3861**	**2797**
VAC079	1	25	**N**	**N**	**N**	**15**	**6**		**25**		**34**		**52**		**70**		**166**		**429**		**306**
VAC079	1	22	**N**	**N**	**N**	**N**	**5**		**18**		**23**		**100**		**60**		**243**		**568**		**793**
VAC079	1	27	**1**	**N**	**N**	**N**	**27**		**23**		**34**		**28**		**27**		**107**		**49**		**57**
VAC079	1	23	**N**	**N**	**7**	**N**	**27**		**6**		**40**		**40**		**151**		**194**		**370**		**494**

Trial	Group	DoD	D18.5	D19	D19.5	D20	D20.5	D21	D21.5	D22	D22.5	D23	D23.5	D24	D24.5	D25	D25.5	D26	D26.5	D27	D27.5
VAC069	9	15.5																			

VAC069	9	18	**20348**																		
VAC069	9	19	**9432**	**19781**																	
VAC069	9	14																			
VAC069	9	16																			
VAC069	9	14.5																			
VAC069	9	15																			
VAC079	1	19	**3769**	**13066**	**10752**																
VAC079	1	19	**6564**	**12971**																	
VAC079	1	25		**968**		**745**		**2043**	**2019**	**3302**	**2464**	**4198**	**4425**	**6010**	**5591**	**15486**	**10533**				
VAC079	1	22		**2504**	**2008**	**3138**	**3749**	**8451**	**7364**	**12330**	**9359**										
VAC079	1	27		**86**		**173**		**397**		**565**		**930**		**1597**		**3766**	**3290**	**5621**	**5404**	**12220**	**8699**
VAC079	1	23		**1242**		**1338**		**3417**		**4662**	**7714**	**13665**	**13903**								

Table S8. Malaria qPCR data (gc/mL) for CHMI in October 2021. Malaria qPCR data used in PMR modelling. Top row represents day (D) of follow-up visit post blood-stage CHMI. DoD indicates the timepoint at which malaria diagnostic criteria was reached. VAC069 Group 12, infectivity controls; VAC071 Group 2, VV-PvDBPII given at 0, 17, 19 months; VAC079 Group 2, PvDBPII/M-M given at 0, 1, 2 months. Treatment in some volunteers was started half a day after reaching malaria diagnostic criteria. qPCR data shown for samples taken prior to starting treatment. qPCR negative values for all three triplicate readings in the assay are indicated by 'N'. Squares highlighted in gray indicate negative or < 20 gc/mL which is below minimum positive reporting criteria and these datapoints were removed for PMR modelling. Blacked out boxes indicate the timepoints after a volunteer commenced antimalarial treatment.

Trial	Group	DoD	D7	D8	D9	D10	D11	D11.5	D12	D12.5	D13	D13.5	D14	D14.5	D15	D15.5	D16	D16.5	D17	D17.5
VAC069	12	15	**8**	**28**	**66**	**124**	**604**		**806**		**2823**	**2487**	**3759**	**3551**	**14373**	**10759**				
VAC069	12	15	**17**	**17**	**71**	**119**	**1021**	**804**	**1617**	**1727**	**3405**	**4145**	**5742**	**6828**	**17155**	**13910**				
VAC069	12	17	**N**	**9**	**N**	**7**	**73**		**46**		**510**		**501**		**1735**	**1792**	**3600**	**4258**	**12302**	**13366**
VAC069	12	15.5	**N**	**61**	**53**	**73**	**401**		**767**		**1552**	**1096**	**2597**	**2245**	**5737**	**3057**				
VAC071	2	13	**47**	**54**	**128**	**279**	**1252**	**1196**	**2498**	**2221**	**9116**	**10400**								
VAC071	2	15	**N**	**28**	**81**	**162**	**447**		**950**		**1941**	**1523**	**2321**	**1996**	**6614**					
VAC071	3	16	**27**	**60**	**73**	**107**	**620**		**912**		**1918**	**2059**	**2644**	**6468**	**7675**	**4376**	**12306**	**24606**		
VAC071	3	14.5	**N**	**70**	**76**	**219**	**517**		**885**		**3010**	**3061**	**5275**	**6615**						
VAC071	3	17	**9**	**N**	**17**	**39**	**123**		**102**		**400**		**787**		**2660**	**1875**	**4213**	**5979**	**15424**	**16420**
VAC079	2	14.5	**17**	**20**	**169**	**242**	**1227**	**989**	**1517**	**2934**	**5399**	**6277**	**8780**	**17594**	**33726**					
VAC079	2	14.5	**N**	**107**	**129**	**90**	**1244**	**852**	**1657**	**1751**	**2228**	**5138**	**8719**	**11292**	**42914**					
VAC079	2	15	**9**	**14**	**61**	**46**	**536**		**652**		**1961**	**2072**	**2070**	**1030**	**10301**	**7374**				
VAC079	2	16	**8**	**21**	**130**	**65**	**503**		**611**		**1855**	**1765**	**2681**	**4131**	**7432**	**5408**	**13620**	**21801**		

Table S9. Summary of PMR analysis. *Two tailed *p* value reported for Mann-Whitney test comparing controls with each vaccine group.

	Controls	VV-PvDBPII	PvDBPII/M-M (0,1,2m)	PvDBPII/M-M (0,1,14m)
No of volunteers	13	8	4	6
Median PMR per 48 h	6.8	5.4	6.3	3.2
Range PMR per 48 h	4.0 to 11.1	4.0 to 7.3	5.1 to 7.9	2.3 to 4.3
D'Agostino & Pearson K^2 test	$p = 0.02$	$p = 0.78$	$p = 0.57$	
Mann-Whitney test*		$p = 0.14$	$p = 0.01$	

Table S10. Analysis of PMR by study group and Duffy blood group serophenotype. Multiple linear regression was used to test if PMR differed significantly between different Duffy blood group antigen (DARC) serophenotypes after controlling for vaccination group.

	Univariate predictor			Adjusted for other variable		
Variable	Estimate	95% CI	*p* value	Estimate	95% CI	*p* value
Intercept	6.6	5.8 to 7.4	<0.001	6.3	5.5 to 7.2	<0.001
Study group						
Controls	0			0		
VV-PvDBPII	-1.1	-2.4 to 0.2	0.08	-1.2	-2.5 to 0.2	0.08
PvDBPII/M-M (0,1,2m)	-0.2	-1.9 to 1.4	0.76	0.04	-1.6 to 1.7	0.96
PvDBPII/M-M (0,1,14m)	-3.4	-4.8 to -2.0	<0.001	-3.3	-4.7 to -1.9	<0.001
DARC serophenotype						
Fya^+Fyb^+				0		
Fya^+Fyb^-				1.2	-0.1 to 2.6	0.08
Fya^-Fyb^+				0.02	-1.2 to 1.3	0.97
R squared	0.49			0.56		
No observations	31			31		
